# High-gradient magnetic separation chip–based small extracellular vesicle isolation for noninvasive subtyping of primary aldosteronism

**DOI:** 10.1126/sciadv.aeb4949

**Published:** 2026-04-17

**Authors:** Dong Wang, Jun Zhou, Zhixin Chen, Junyuan Yang, Yingjie Li, Shiwei Sun, Dongxu Qiu, Yao Wang, Yushi Zhang, Mingzhu Yang

**Affiliations:** ^1^ Department of Urology, Peking Union Medical College Hospital, Chinese Academy of Medical Sciences and Peking Union Medical College, Beijing 100730, China.; ^2^ Core Instrument Facility, Institute of Basic Medical Sciences, Chinese Academy of Medical Sciences and Peking Union Medical College, Beijing 100005, China.; ^3^ Beijing Institute of Technology, Beijing 100081, China.; ^4^ Beijing Institute of Mechanical Equipment, Beijing 100143, China.

## Abstract

Primary aldosteronism (PA), a leading cause of secondary hypertension, remains vastly underdiagnosed due to unreliable screening tools. We developed an integrated platform combining a high-gradient magnetic separation three-dimensional (HGMS-3D) chip and locked nucleic acid (LNA)–enhanced droplet digital polymerase chain reaction (ddPCR) for noninvasive detection of potassium inwardly rectifying channel subfamily J member 5 (*KCNJ5*) mutations in plasma small extracellular vesicles (sEVs). The HGMS-3D chip uses a nickel mesh–based stereoscopic immunoaffinity capture system, achieving a vesicle isolation efficiency 4.4-fold higher than ultracentrifugation. Coupled with LNA-ddPCR, the platform detects *KCNJ5* hotspot mutations [p.Gly151Arg, (G151R); p.Leu168Arg (L168R)] at minor allele frequencies of ≤0.05% (*R*^2^ ≥ 0.99), overcoming plasma-derived noise. Clinical validation in 106 patients with PA demonstrated 64.58% sensitivity and 96.88% specificity for sEV-based mutation profiling. The assay identified one aldosterone-producing adenoma (APA) case missed by tissue genotyping, achieving area under the receiver operating characteristic curve (AUC) values of 0.767 ~ 0.852 across mutations. This noninvasive approach could enable curative treatment for millions with undiagnosed PA, advancing precision management of endocrine hypertension through sEV-based liquid biopsy.

## Teaser

A noninvasive platform detects key mutations in plasma vesicles to identify underdiagnosed primary aldosteronism.

## INTRODUCTION

Primary aldosteronism (PA) is an endocrine abnormality caused by overproduction of aldosterone from the diseased adrenal gland, inducing inappropriate sodium retention and increased blood volume, thus leading to hypertension, the major cardiovascular risk factor (*1*). In recent years, PA has been found responsible for the most common cause of secondary hypertension, affecting more than 20% of the population with severe/refractory hypertension (*2*). However, the clinical recognition of PA is extremely poor due to lack of awareness and identifiable clinical features, also suggesting a serious underestimation of the prevalence (*3*, *4*). Patients with PA carry at least a twofold higher risk of cardiovascular diseases compared to patients with essential hypertension (*5*, *6*). Although PA has shown an undoubted contribution to global cardiovascular morbidity, less than 1% of the patients with PA are diagnosed and treated in clinical practice (*6*–*8*). In particular, the treatment of PA demonstrates extremely high cure results, and early diagnosis and subtyping also provide a more substantial prognosis (*5*, *9*). Therefore, accurate and efficient methods for detecting PA are urgently needed to identify the large number of patients who remain undiagnosed to reduce the global burden of hypertension and especially to address this pervasive diagnosis gap.

The elevated plasma aldosterone-renin activity ratio (ARR) is commonly used as a qualitative indicator for the clinical diagnosis of PA (*10*). Although this two-variable ratio is simple and reproducible, its diagnostic performance is easily affected by antihypertensive medications as well as serum potassium levels, yielding false-positive rates as high as 30% and necessitating costly confirmatory testing (*2*, *9*, *11*, *12*). To maximize accuracy, one or more dynamic confirmatory tests (e.g., saline infusion and captopril challenge) are recommended to demonstrate aldosterone overproduction that is nonsuppressible, but they are still criticized for lacking standardization across centers, with sensitivity ranging from 70 to 95% (*6*, *12*). Furthermore, subtype differentiation is the most critical purpose of PA diagnosis, mainly including aldosterone-producing adenomas (APAs) and bilateral adrenal hyperplasia (IHA), as it determines the best treatment at both extremes (*13*). Patients with APA can completely recover from hypertension and markedly reduce cardiovascular risk with unilateral adrenalectomy, in contrast to patients with IHA, for whom unilateral adrenalectomy is typically ineffective and lifetime mineralocorticoid receptor antagonist therapy is the recommended treatment (*7*, *14*). However, none of the subtype identification options used in the clinic could satisfy the request to accurately and easily identify surgically curable APAs. As adrenal computed tomography (CT) scans are unable to accurately identify obscure lesions, adrenal vein sampling (AVS) has been used as the gold standard for clinical subtyping, providing definitive functional evidence to guide unilateral adrenalectomy (*12*, *15*). However, as an invasive and technically demanding procedure requiring specialized expertise, AVS is available in only a limited number of medical centers globally, resulting in diagnostic delays or misclassification (*16*). Even when performed, AVS shows a 10% risk of failure, with complications including adrenal hemorrhage and radiation exposure (*17*). Fortunately, sequencing of a large number of tumor tissues made a important finding that the potassium inwardly rectifying channel subfamily J member 5 (*KCNJ5*) gene mutations, mainly p.Gly151Arg (G151R) and p.Leu168Arg (L168R) occur frequently in APA (*18*, *19*). As the most common genetic variant in APA, the prevalence of *KCNJ5* gene mutations is up to 43% in Europe and 80% in Asian countries (*19*, *20*). Thus, we believe that *KCNJ5* gene mutations can be used as biomarkers to identify APA and IHA in PA. Although mutation analysis of adrenal tissue provides a definitive diagnosis, it is unsuitable for large-scale screening. Therefore, a noninvasive method to determine *KCNJ5* status would enable the efficient identification of patients whose hypertension is potentially curable by unilateral adrenalectomy. Liquid biopsy via analysis of small extracellular vesicles (sEVs) presents a promising strategy to obtain such genetic insights without the need for invasive tissue sampling.

Exosomes, sEVs of ~30 to 150 nm in diameter, originate in the endosomal pathway and are secreted into body fluids, thus inheriting the biomolecular array of the parent cells, making them ideal biomarkers for liquid biopsies (*21*, *22*). As they carry genetic alterations in the molecular landscape of tumors, sEVs can replace cells to provide direct pathological information such as gene mutations, hence avoiding invasive biopsies (*23*, *24*). Due to the bilayer lipid membrane structure, sEVs can reduce the fragmentation of mutant genes significantly compared to circulating tumor DNA, improve the signal-to-noise ratio, and robustly increase the sensitivity of mutation detection (*25*, *26*). Assessing the mutation status of the *KCNJ5* gene in sEVs has provided valuable ideas for the early diagnosis and subtyping of PA. However, the low concentration and nanoscale of sEVs make isolation and collection of sEVs from complex biological matrices an extremely challenging task (*27*, *28*). Although ultracentrifugation (UC) is currently recognized as the “gold standard” for the isolation of sEVs, it is laborious and time-consuming, while unstable recoveries limit the potential application of UC (*29*). Furthermore, due to the enormous centrifugal force, sEVs are susceptible to physical damage and cleavage, which lead to loss of biological information (*30*, *31*), interfering with the accuracy of detection. Polymer precipitation has been used to simplify the sEV extraction process, but coprecipitation of nonvesicular contaminants severely affects sEVs purity (*32*, *33*). Currently, magnetic bead– or microfluidic-based immunoaffinity capture provides unprecedented enrichment of sEVs with high purity and biological activity, whereas the isolation efficiency is still poor (*34*–*36*). If technical problems such as long time-consuming and poor capture efficiency can be solved, it will greatly improve the prospect of sEV-based disease diagnosis for clinical applications.

Here, we present a high-gradient magnetic separation three-dimensional (HGMS-3D) chip–based platform for highly efficient isolation of sEVs, while combining droplet digital polymerase chain reaction (ddPCR) to address the unmet clinical need for noninvasive and precise diagnosis of APA, a surgically curable subtype of PA. The HGMS-3D chip–based platform integrates a 3D nickel mesh–based magnetic architecture, dispersedly distributed immunocapture beads, and stereoscopic chamber for isolation and enrichment of sEVs, achieving 4.4-fold higher than conventional UC via high-frequency collision-mediated immunoaffinity interactions. Locked nucleic acid (LNA)–enhanced ddPCR probes are capable of detecting *KCNJ5* mutations at minor allele frequencies (MAFs) of <0.05%, overcoming the noise limitations of plasma-derived sEVs. Validated in 106 PA patient plasma samples, the platform demonstrated robust clinical performance with 64.58% sensitivity and 96.88% specificity for *KCNJ5* mutation detection from sEVs, achieving concordance with tissue genotyping while resolving previously undiagnosed PA subtypes. By establishing the first sEV-based workflow for *KCNJ5* mutation profiling, this study provides an innovative, noninvasive tool to identify potential APA patients. This approach holds potential for large-scale screening within the diagnostic pathway, potentially helping to prioritize patients for definitive subtyping and curative treatment.

## RESULTS

### Principle of the HGMS-3D chip for isolation and analysis of sEVs

**Fig. 1.**
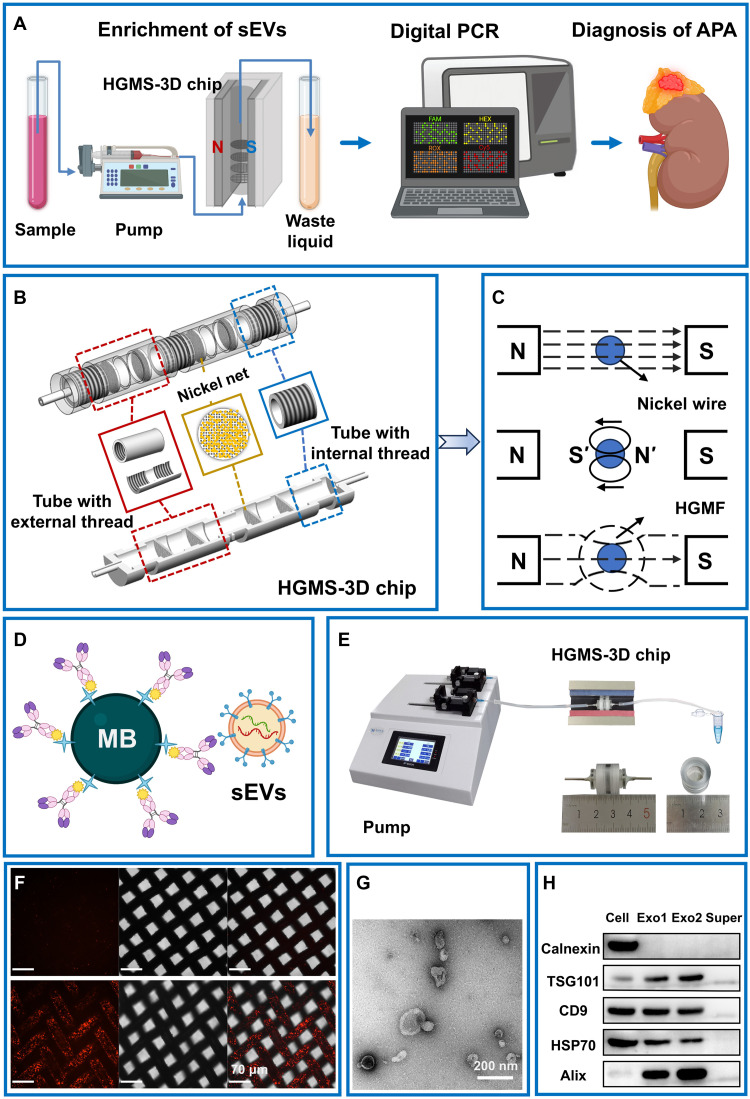
Overview of the HGMS-3D chip designed for sEV isolation and detection. (**A**) Flowchart of sEV isolation from PB samples for APA diagnosis. (**B**) Schematic representation of the internal structure of HGMS-3D chip. (**C**) Schematic illustration of high-gradient magnetic field generation within the HGMS-3D chip. (**D**) Schematic representation depicting the specific capture of sEVs by immunomagnetic beads conjugated with CD63 and CD9 antibodies. MB, magnetic bead core. (**E**) Physical representation of each component of the HGMS-3D chip in series. (**F**) Characterization of sEVs from the supernatant of 293T cells on the HGMS-3D chip using laser confocal microscopy. Scale bars, 70 μm. (**G**) Morphological analysis of sEVs in the supernatant of 293T cells subsequent to extraction from the HGMS-3D chip via electron microscopy. Scale bar, 200 nm. (**H**) Western blots of calnexin, TSG101, CD9, HSP70, and Alix for sEVs separated from two different patients (Exo1 and Exo2). 293T cells (cell) and culture supernatant (super) served as controls.

To establish a system for noninvasive diagnosis of APA, we developed a platform that combines the HGMS-3D chip and ddPCR. The HGMS-3D chip is designed for effective isolation of sEVs, while ddPCR is used for the detection of gene mutations. Plasma samples from patients with APA are processed with the HGMS-3D chip to capture sEVs, and the enriched sEVs are recovered for downstream detection of *KCNJ5* gene mutations, enabling diagnosis of APA (Fig. 1A).

The HGMS-3D chip consists of a 3D chamber with multilayer nickel meshes distributed inside, an external magnetic circuit, and a liquid-control system. Immunomagnetic beads for the capture of sEVs are anchored on the 3D-arranged nickel meshes under an external magnetic field, forming hundreds of millions of capture sites with a stereoscopically dispersed distribution (Fig. 1B). The magnetic circuit provides an external magnetic field to magnetize the multilayer nickel meshes and generate a high-gradient magnetic field (HGMF) around each nickel wire, enabling attraction of immunomagnetic beads throughout the entire isolation period (Fig. 1C). Magnetic beads coated with CD9 and CD63 antibodies are prepared via a reaction between streptavidin and biotin for immunoaffinity reaction–mediated isolation of sEVs (Fig. 1D). The liquid-control system, composed of an injection pump and pipelines, delivers continuous liquid flow to complete sample processing (Fig. 1E). After processing, the HGMS-3D chip is disassembled to collect the nickel meshes and recover sEVs. To avoid loss of sEVs and nucleic acids, sEVs are directly lysed while still bound to the nickel meshes and immunomagnetic beads. Subsequently, the extracted DNA is detected and analyzed to obtain information on *KCNJ5* gene mutations.

To characterize the sEVs captured by the HGMS-3D chip, we collected sEVs from the supernatant of 293T cells by UC and labeled them with Paul Karl Horan 26 (PHK26). We then recaptured the diluted sample of labeled sEVs with the HGMS-3D chip, retrieved the nickel meshes, and observed them under a fluorescence microscope. In the bright-field image, the nickel meshes exhibited a grid-like structure with nickel wires (average diameter of 27.4 μm) and holes (average diameter of 30.8 μm). In the fluorescence and merged images, red fluorescence was observed along the nickel wires but was absent in the control group (dilution buffer only), indicating the aggregation of PKH26-stained sEVs on the nickel meshes (Fig. 1F). The sEVs captured by the HGMS-3D chip were released by treatment with 50 mM dithiothreitol and recovered for morphological characterization by transmission electron microscopy (TEM). TEM revealed typical morphological features of sEVs, which were round to oval vesicles with intact membranes, ~20 to 80 nm in diameter, and containing material of low electron density (Fig. 1G). Western blot analysis was performed to determine the expression of four common exosomal proteins in the treated samples. Plasma samples from two different patients (Exo1 and Exo2) and the cell-culture supernatant of 293T cells showed the presence of the sEV-specific marker proteins tumor susceptibility gene 101 protein (TSG101), CD9, heat shock protein 70 (HSP70), and apoptosis-linked gene 2-interacting protein X (Alix), while the cytoplasmic protein calnexin was absent (Fig. 1H).

### Optimization of the HGMS-3D chip for sEV isolation

**Fig. 2.**
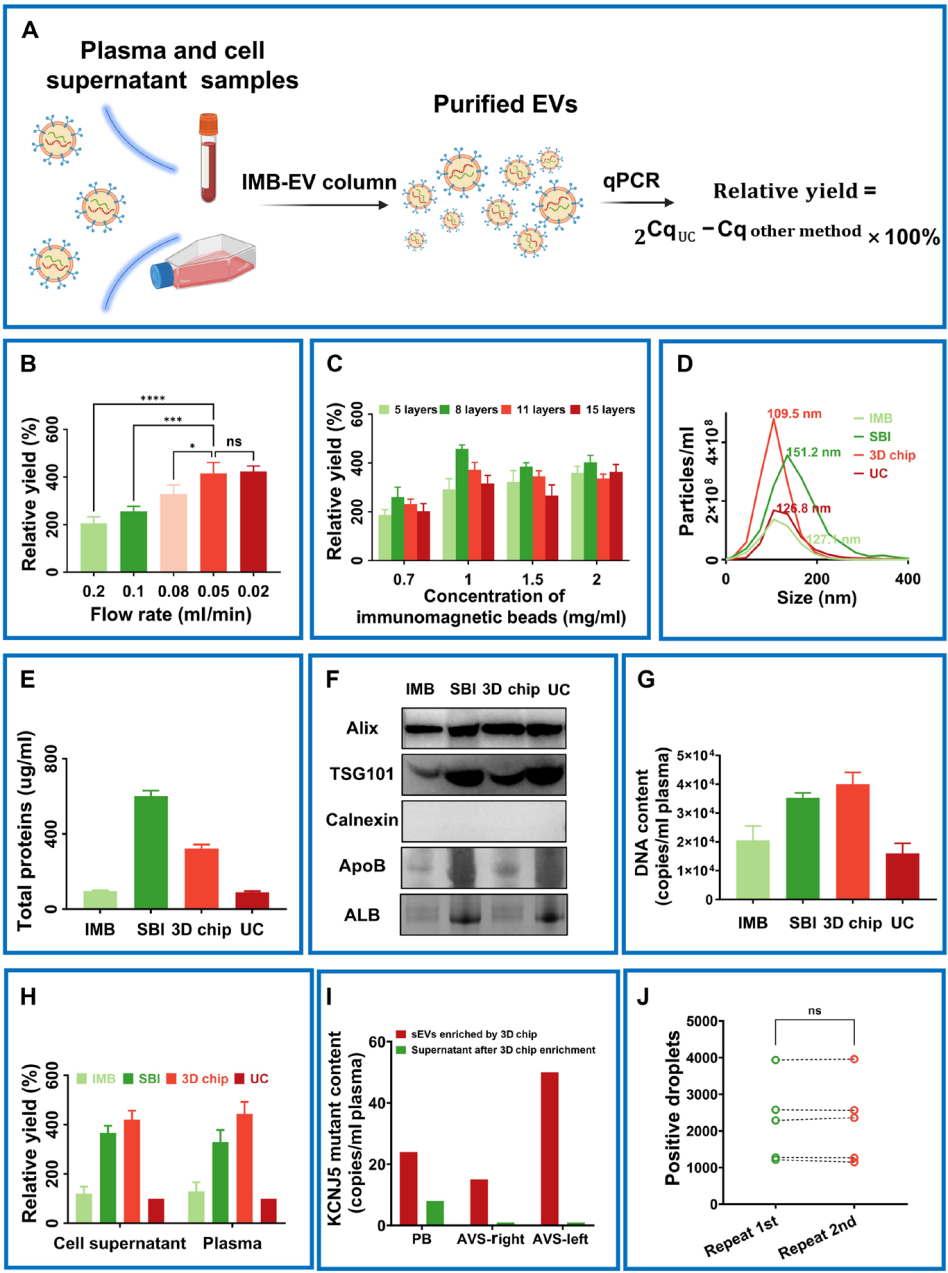
Systematic optimization the HGMS-3D chip for sEV isolation. (**A**) Calculation of the relative yield of the KCNJ5 gene captured by the HGMS-3D chip using qPCR. (**B**) Relative yield of sEVs captured from 1 ml of plasma using the HGMS-3D chip at varying flow rates. (**C**) Relative yield of sEVs captured from 10 ml of 293T cell supernatant using HGMS-3D chips with varying immunomagnetic bead concentrations and nickel mesh layers. (**D**) Particle number and size distribution captured from 1 ml of plasma by the HGMS-3D chip, IMB, SBI, and UC. (**E**) Total protein content of sEVs captured from 1 ml of plasma by the HGMS-3D chip, IMB, SBI, and UC. (**F**) Western blots of TSG101, Alix, and calnexin of sEVs separated by the HGMS-3D chip, IMB, SBI, and UC and plasma contaminants [apolipoprotein B (ApoB) and albumin (ALB)] served as negative controls. (**G**) Total DNA content of sEVs captured from 1 ml of plasma by the HGMS-3D chip, IMB, SBI, and UC. (**H**) Relative yield of the KCNJ5 gene for sEVs captured from 10 ml of 293T cell culture supernatant and 1 ml of plasma by the HGMS-3D chip, IMB, SBI, and UC. (**I**) *KCNJ5* mutation content in exosomes and supernatants separated by HGMS-3D chip from 1 ml of peripheral and left/right AVS plasma. (**J**) Reproducibility of HGMS-3D chip for capturing sEVs (data are expressed as the means ± SD of three independent analyses). ns, not significant.

To quantify the isolation efficiency of the HGMS-3D chip for sEVs, we extracted DNA from the purified sEVs and performed quantitative PCR (qPCR) to detect the *KCNJ5* gene. The relative yield of the HGMS-3D chip was calculated by comparing the *KCNJ5* gene levels in sEVs enriched using the HGMS-3D chip with those obtained via UC (Fig. 2A).

To study the influence of flow rate, immunomagnetic bead concentration, and nickel mesh layers on relative yield, we first optimized the flow rate for sEV capture using the HGMS-3D chip. The relative yield of captured sEVs was determined across flow rates ranging from 0.02 to 0.2 ml/min. Relative yield increased as flow rate decreased; however, no significant difference was observed between 0.05 and 0.02 ml/min. Balancing yield and isolation efficiency, we selected 0.05 ml/min for plasma sEV capture (Fig. 2B). Next, we optimized the immunomagnetic bead concentration and nickel mesh layer count. The highest relative yield was achieved at a bead concentration of 1 mg/ml and eight layers of nickel mesh (Fig. 2C).We further evaluated the HGMS-3D chip by comparing particle size distribution, particle count, protein content, and relative yield of sEVs enriched from cell supernatants and plasma with UC, immunomagnetic bead–based isolation (IMB), and the System Biosciences ExoQuick kit (SBI). Nanoparticle tracking analysis (NTA) characterized particle size distribution and count, while protein content was quantified using a bicinchoninic acid (BCA) assay. The HGMS-3D chip yielded the highest particle count (1.3 × 10^9^ particles/ml), followed by SBI, IMB, and UC. It also captured sEVs with the smallest average size (109.5 nm) and narrowest size distribution (Fig. 2D). The mean size determined by NTA was larger than that characterized by TEM, as NTA measures the hydrodynamic diameter of particles in suspension, while TEM visualizes the dehydrated core structure after sample fixation and staining, which often results in a smaller apparent size. Notably, although the IMB method and the HGMS-3D chip used immunomagnetic beads functionalized with the same antibodies, the unique 3D architecture and flow dynamics of the HGMS-3D chip resulted in its markedly superior performance. Total protein quantification showed that SBI yielded the highest protein content, followed by the HGMS-3D chip, IMB, and UC (Fig. 2E). In the Western blot analysis, isolation by the HGMS-3D chip confirmed the presence of the sEV-specific markers TSG101 and Alix, while the negative control calnexin was absent (Fig. 2F). Crucially, analysis of common plasma contaminants revealed markedly lower levels of apolipoprotein B and albumin in the HGMS-3D chip isolates compared to other methods, particularly SBI, highlighting its superior specificity. The HGMS-3D chip achieved the highest total DNA yield from plasma sEVs (Fig. 2G). The lower plasma contaminants and higher particle count in HGMS-3D chip eluate compared to other methods, coupled with markedly higher DNA content, indicate superior purity. Furthermore, the markedly higher yield compared to the conventional IMB methods further validates the critical role of the chip’s 3D capture matrix in collision-enhanced binding efficiency. Under optimal conditions of immunomagnetic beads (1 mg/ml) and an eight-layer nickel mesh, the HGMS-3D chip achieved the highest relative yield for both cell supernatants and plasma. In cell culture supernatant samples, the HGMS-3D chip achieves a vesicle isolation efficiency 4.2-fold greater than UC. In plasma samples, it achieves a vesicle isolation efficiency 4.4-fold higher than UC. (Fig. 2H).

To confirm that the detected DNA was intravesicular, we separately analyzed sEVs and the postcapture supernatant. The result demonstrated that the *KCNJ5* mutation signal was almost exclusively contained within the sEVs fraction, with negligible signal in the supernatant, for the plasma of both peripheral blood (PB) and AVS (Fig. 2I and fig. S1). Last, technical replicate experiments demonstrated high reproducibility of sEVs capture by the HGMS-3D chip, with no significant difference in mutant droplet counts between independent runs (Fig. 2J). Together, these data established that the HGMS-3D chip offers an efficient, reproducible platform for the high-yield and high-purity isolation of sEVs, effectively enriching for vesicle-encapsulated nucleic acids with minimal contamination.

### Detection of *KCNJ5* mutations in isolated sEVs using digital PCR

**Fig. 3.**
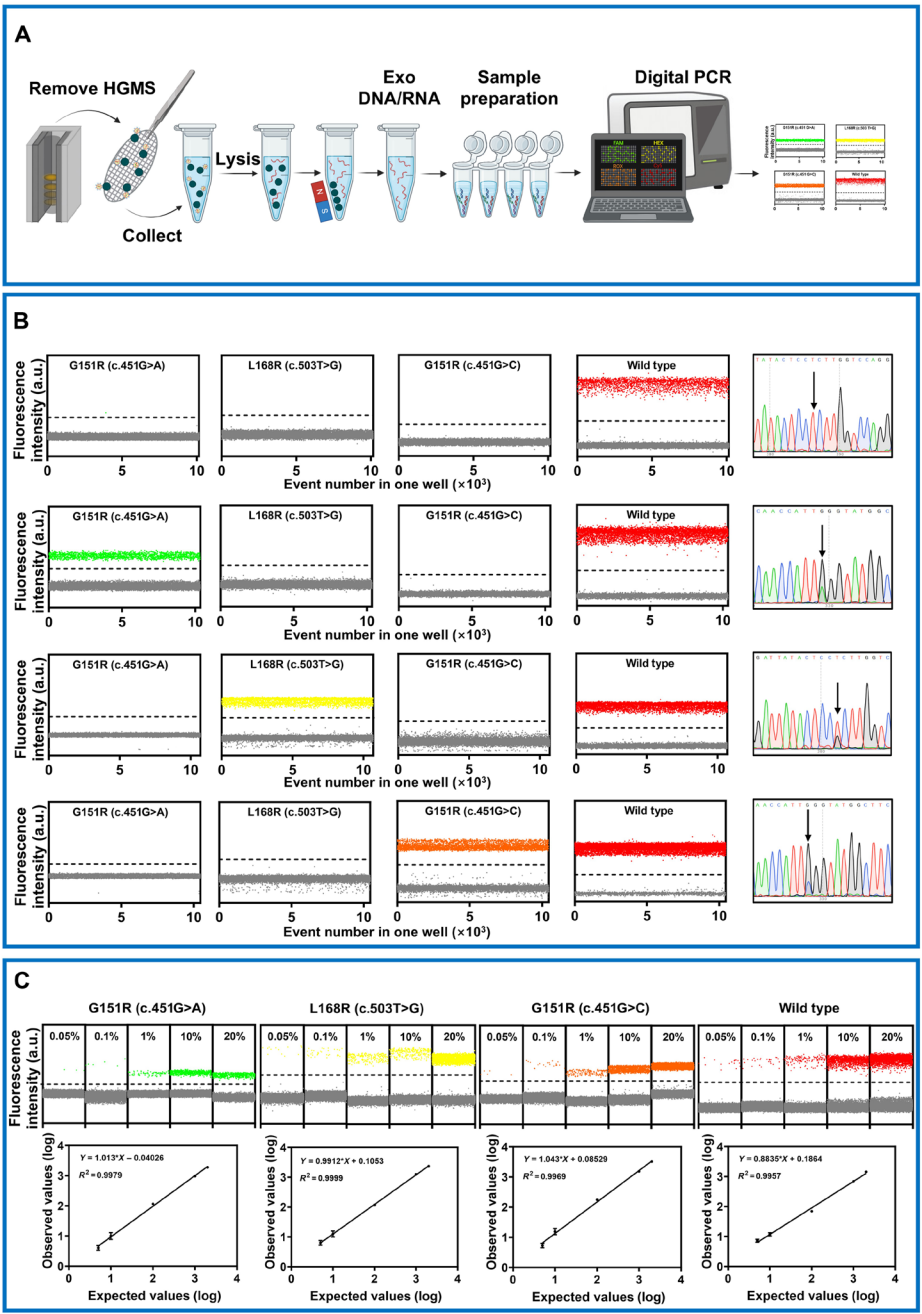
Isolation of sEVs and quantitative performance of ddPCR panels for KCNJ5 mutation analysis. (**A**) Workflow for isolating sEVs using the HGMS-3D chip and subsequent KCNJ5 mutation analysis via ddPCR. (**B**) Specificity of the ddPCR assays. (**C**) Sensitivity and linearity of the ddPCR assays. a.u., arbitrary unit.

To analyze *KCNJ5* gene mutations in isolated sEVs, we established LNA-enhanced ddPCR (LNA-ddPCR). After isolating sEVs using the HGMS-3D chip, the customized magnet was removed, and the nickel mesh was transferred to a centrifuge tube. Lysis buffer was then added to lyse sEVs and release nucleic acids, followed by immediate LNA-ddPCR analysis. Copy numbers of mutant and wild-type (WT) alleles were determined from droplet scatter plots, enabling calculation of the MAF (Fig. 3A). Primer probes were modified with LNA to enhance discrimination between WT and mutant alleles (table S1).

To assess assay specificity, we analyzed three distinct mutation types and WT *KCNJ5* in adrenal adenoma (APA) tissue samples previously characterized by Sanger sequencing. Digital PCR results fully matched Sanger sequencing data. Each assay generated robust mutation-specific signals with clear clustering and minimal cross-reactivity at a 20% mutation frequency (Fig. 3B).

To evaluate sensitivity and linear detection ranges, mutant and WT DNA from patients with APA were mixed and serially diluted against a constant WT genomic DNA background (10,000 copies per reaction), achieving theoretical MAFs of 20, 10, 1, 0.1, and 0.05%. Measured DNA concentrations correlated with expected values across all dilutions (20 to 0.05%). Strong linear correlations existed between measured and expected mutant fractions for all assays (*R*^2^ ≥ 0.99). Background MAF (limit of blank, LoB) was determined using WT DNA from non-PA patients, while limits of detection (LoDs) were established using mutant DNA mixtures. LoBs were 0.02% for G151R (c.451G>A), 0.03% for G151R (c.451G>C), and 0.04% for L168R (c.503T>G), with all LoDs at 0.05% MAF. These assays demonstrated high sensitivity and reliability for detecting low-frequency variants against predominant WT backgrounds (Fig. 3C).

### *KCNJ5* mutation detection of patients with APA using isolated sEVs

To evaluate the clinical performance of our *KCNJ5* mutation detection system (LNA-ddPCR) coupled with HGMS-3D chip–based sEV isolation, we analyzed a cohort of plasma samples alongside matched tumor tissues. The cohort comprised 106 patients with postoperatively confirmed APA [based on CT imaging and Primary Aldosteronism Surgical Outcome (PASO) criteria] and 15 patients diagnosed with IHA (table S2). All plasma samples were processed from 1 ml of plasma stored at −80°C.

**Fig. 4.**
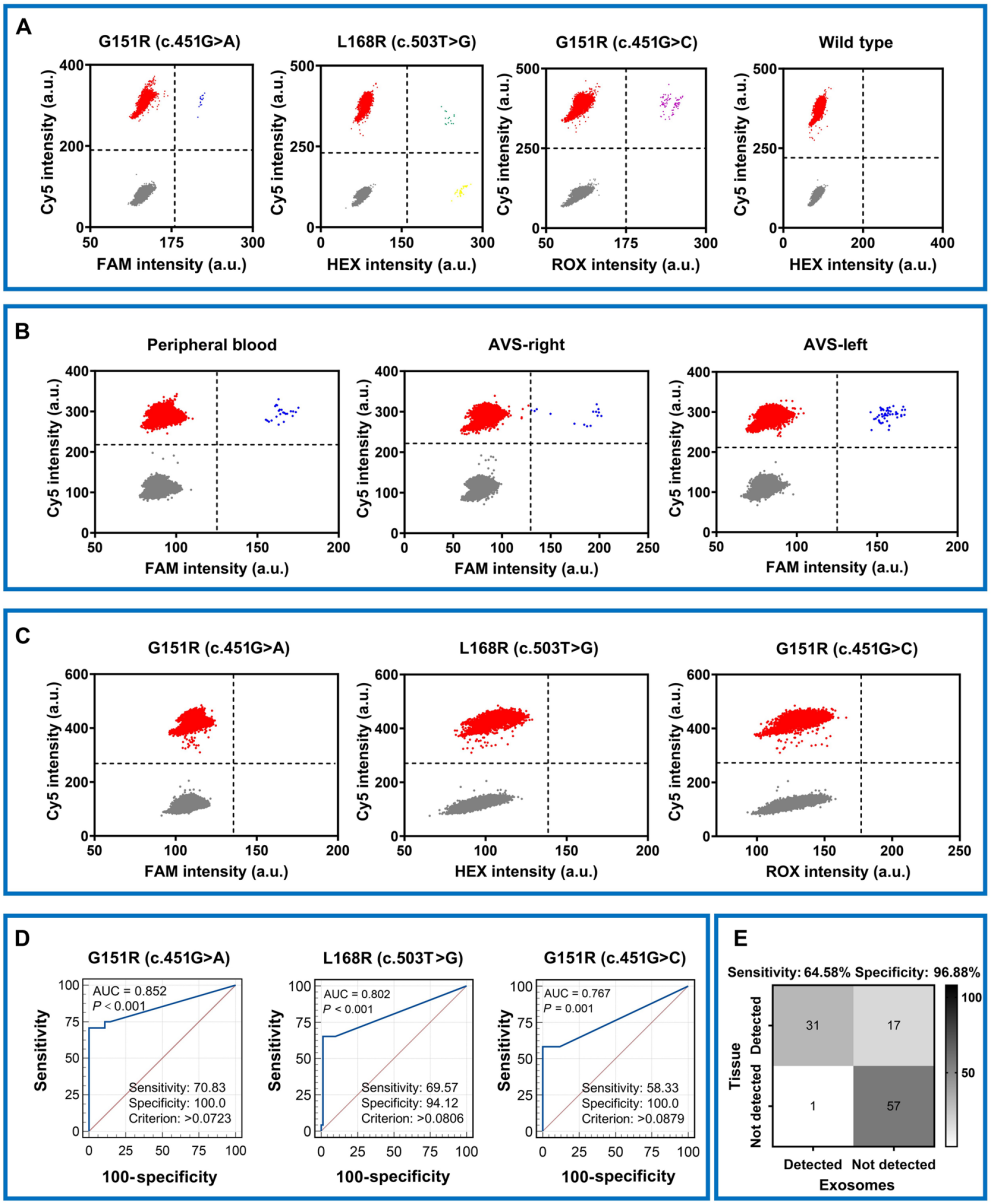
KCNJ5 mutation detection in clinical samples. (**A**) 2D ddPCR plots showing KCNJ5 variants: G151R (c.451G>C), G151R (c.451G>A), L168R (c.503T>G), and WT in sEVs of APA clinical samples. (**B**) 2D ddPCR plots showing G151R (c.451G>A) variants in sEVs of PB, AVS-right, and AVS-left. (**C**) 2D ddPCR plots showing KCNJ5 variants: G151R (c.451G>C), G151R (c.451G>A), and L168R (c.503T>G) variants in sEVs of IHA clinical samples. (**D**) ROC curves for KCNJ5 mutation detection by ddPCR. Optimal cutoff criteria and AUC values are indicated. (**E**) Confusion matrix comparing total KCNJ5 mutation status in sEVs versus tissue. Sample counts are shown in each quadrant.

In the assessment of the mutation status in the tumor tissues of the 106 plasma samples, 59 patients exhibited mutations in the *KCNJ5* gene. Specifically, the number of patients who presented the G151R mutation (c.451G>A), the G151R mutation (c.451G>C), and the L168R mutation (c.503T>G) is 24, 12, and 23. Next, we detected the *KCNJ5* gene mutation status in the sEVs from plasma of the 106 patients with APA. The representative 2D plots of *KCNJ5* mutants were shown in (Fig. 4A).

To explore the potential of our assay in a sample type enriched for adrenal-derived vesicles, we analyzed matched PB and AVS specimens from a patient with *KCNJ5* mutation–positive APA. The mutant allele frequency was markedly higher in sEVs isolated from the left AVS sample compared to the PB, while the right AVS sample showed substantially lower levels (Fig. 4B). This result demonstrated that the affected adrenal vein carries a higher concentration of mutant sEVs, while PB sEVs average the content from both adrenal veins. This finding serves as a compelling proof of concept for the biological validity of our approach. Analysis of plasma sEVs from all 15 patients with IHA yielded negative results for all three *KCNJ5* hotspot mutations (Fig. 4C and table S3).

Receiver operating characteristic (ROC) curves to determine *KCNJ5* mutational status from MAFs were generated. In 106 patients with APA, the *KCNJ5* gene G151R (c.451G>A) mutation in plasma sEVs was detected in 17 of 24 tumor tissue–positive samples and not detected in seven samples. Of the 82 negative samples, none was detected as positive. The detection of this site achieved an accuracy of 93.34% (99 of 106), a specificity of 100% (17 of 17), and a sensitivity of 70.83% (17 of 24), with an area under the ROC curve (AUC) of 0.852 [95% confidence interval (CI), 0.770 to 0.914]. In 106 patients with APA, the *KCNJ5* gene G151R (c.451G>C) mutation in plasma sEVs was detected in 7 of 12 tumor tissue–positive samples, and 5 were not detected. Of the 93 negative samples, 93 were detected as negative. The detection of this site achieved an accuracy of 94.33% (100 of 106), a specificity of 100% (7 of 7), and a sensitivity of 58.3% (7 of 12), with an AUC of 0.767 (95% CI, 0.674 to 0.844). In 106 patients with APA, the *KCNJ5* gene L168R (c.503T>G) mutation in plasma sEVs was detected in 16 of 23 positive samples, while seven were not detected. Among the 82 negative samples, one was detected and the other 81 were negative. The detection of this site achieved an accuracy of 91.51% (97 of 106), a specificity of 94.12% (16 of 17), and a sensitivity of 69.57% (16 of 23), with an AUC of 0.802 (95% CI, 0.713 to 0.873) (Fig. 4D). The *KCNJ5* total mutation detection rate of sEV DNA showed 64.58% sensitivity and 96.88% specificity (Fig. 4E).

## DISCUSSION

PA represents a yet surgically curable but vastly underdiagnosed cause of hypertension, largely due to the invasiveness and limited accessibility of current subtyping methods like AVS (*12*, *37*). To address this unmet need, we established an integrated platform for noninvasive detection of *KCNJ5* mutations in plasma-derived sEVs. This approach synergizes a HGMS-3D chip for efficient sEV isolation and LNA-ddPCR for ultrasensitive mutation profiling. The HGMS-3D chip achieves an exceptional sEV recovery efficiency of 4.4-fold higher than UC, while maximizing recovery rate of nucleic acid and minimizing co-isolation of contaminants. When coupled with LNA-ddPCR, the platform detects *KCNJ5* hotspot mutations (G151R/L168R) at MAFs as low as 0.05% (*R*^2^ ≥ 0.99), overcoming the background noise typical of plasma-derived biospecimens. Clinical validation across 106 patients with PA demonstrated robust diagnostic accuracy (sensitivity: 64.58%; specificity: 96.88%) and identified APA cases missed by tissue genotyping (AUCs: 0.767 to 0.852), offering a transformative tool for precision subtyping. Because this platform cannot provide lateralizing information, it is therefore not a substitute for AVS as the definitive diagnostic standard for determining surgical candidacy. Instead, a positive result offers strong molecular evidence for the presence of an APA. In patients with consistent clinical presentations, this approach can inform and guide treatment decisions, especially when AVS is contraindicated, unsuccessful, or unavailable. Thus, this work establishes a noninvasive, sEV-based method that can contribute to the preoperative stratification of PA by identifying a genetically defined subset of APAs.

We developed a stereoscopic immunoaffinity device, the HGMS-3D chip, to overcome the dual challenges of low recovery efficiency and impurity co-isolation that plague conventional EV isolation methods. This chip leverages a multilayered nickel mesh architecture magnetized to generate high-gradient fields to firmly anchor immunomagnetic beads, thereby creating a 3D matrix with hundreds of millions of stereoscopically dispersed capture sites (anti-CD9/CD63). The core advantage of this design lies in its ability to facilitate continuous and high-frequency collisions between sEVs in the flowing sample and the immobilized capture beads, maximizing binding opportunities and efficiency. This mechanism enables the chip to reconcile high yield with high purity, which is a critical requirement in sEV isolation. This capability is unequivocally demonstrated in comparative studies. Systematic optimization identified key parameters, a flow rate of 0.05 ml/min, an immunomagnetic bead concentration of 1 mg/ml, and an eight-layer nickel mesh, that maximized capture efficiency (Fig. 2, B and C). Comparative analysis revealed the superior performance of the 3D chip, that it achieved the highest particle count (1.3 × 10^9^ particles/ml) and the highest total DNA yield from plasma sEVs, while maintaining a narrow vesicle size distribution centered at 109.5 nm (Fig. 2, D and G). Crucially, it balanced this high yield with exceptional purity. Plasma contamination analysis confirmed that the HGMS-3D chip isolates contained markedly lower levels of nonvesicular impurities compared to SBI and IMB, while preserving sEV integrity markers like TSG101 and Alix (Fig. 2, E, F). This performance markedly outperformed the conventional IMB method using identical antibodies, underscoring the critical role of the chip’s 3D architecture and flow-driven collision dynamics in enhancing binding efficiency (Fig. 2G). The HGMS-3D chip yielded EVs with a smaller mean diameter and narrower size distribution compared to UC, polymer precipitation, and conventional immunocapture (Fig. 2D). While large EVs (lEVs) may also express these tetraspanins, their surface marker profile and biophysical properties differ, potentially leading to their under-representation in our captures. Currently, there is no evidence to suggest that lEVs harbor a greater fraction of *KCNJ5* or other driver mutations in APA, and our data confirm that sEVs alone provide a clinically informative signal. Should future studies indicate a diagnostic benefit in capturing the full EV spectrum, the modular design of our chip allows for the integration of additional antibodies targeting lEV-specific surface markers. Furthermore, a key control experiment demonstrated that the detected *KCNJ5* mutation signal was almost exclusively contained within the sEV fraction, with negligible signal in the postcapture supernatant (Fig. 2I), confirming the intravesicular origin of the mutant DNA and validating our approach. Last, technical replicate experiments established the high reproducibility of the isolation process (Fig. 2J). Collectively, these data establish the HGMS-3D chip as a robust and efficient platform for enriching nucleic acid–rich sEVs with minimal co-isolation of contaminants. By resolving the yield-purity dichotomy, our device establishes a higher standard for sEV isolation—enabling reliable acquisition of biomarker-rich vesicles essential for sensitive liquid biopsy applications.

To detect *KCNJ5* mutations in the isolated sEVs, we used ddPCR, a technology uniquely suited for analyzing fragmented, low-abundance nucleic acids typical of exosome DNA (*38*, *39*). The sensitivity of mutation detection was further enhanced by leveraging LNA-modified ddPCR probes. The partitioning capability of ddPCR allows absolute quantification of fragmented nucleic acids within sEVs, while LNA chemistry improves allelic discrimination through increased melting temperature differences. This combination achieved a detection limit of 0.05% MAF with excellent linearity (*R*^2^ ≥ 0.99; Fig. 3C), which is essential for analyzing tumor-derived DNA diluted in circulation. We validated this integrated workflow in a clinically well-characterized cohort of 106 postoperatively confirmed APA patients and 15 patients with IHA. The assay demonstrated high specificity (96.88%) and an overall sensitivity of 63.8% for detecting tissue-confirmed *KCNJ5* mutations (Fig. 4E). Sensitivity varied by mutation subtype (G151R c.451G>C: 58.33%, G151R c.451G>A: 70.83%, and L168R: 69.57%), a finding that may reflect both technical aspects of probe hybridization and the biological heterogeneity of sEV secretion and DNA packaging from different tumor clones. The LNA-ddPCR assay has a defined LoD (0.05% MAF); thus, mutant alleles present at frequencies below this threshold in the isolated sEV population would be missed. Furthermore, the inherent biological heterogeneity associated with sEV biogenesis and secretion means that the proportion of mutation-bearing sEVs is inherently variable, posing a challenge for developing highly consistent assays. To further improve sensitivity, future iterations of this platform could (i) use a broader panel of capture antibodies (e.g., including CD81 and epithelial cell adhesion molecule (EpCAM)) to maximize the recovery of heterogeneous sEV populations and (ii) integrate even more sensitive mutation detection technologies, such as next-generation sequencing–based assays optimized for low-input, fragmented DNA. By addressing these factors, the noninvasive subtyping of APA can be made more comprehensive and robust. Analysis of our patient cohort confirmed the phenotype of *KCNJ5*-mutant APAs, which presented with larger tumor size compared to WT tumors. However, among *KCNJ5*-mutant patients, we found no significant correlation between the detectability of the mutation in plasma sEVs and standard clinical parameters such as tumor size, blood pressure, or serum potassium levels. This suggests that the current sensitivity of our assay is not primarily limited by the gross clinical severity of the disease. Instead, the failure to detect all mutation-positive cases likely stems from biological factors, such as heterogeneous sEV release and variable mutant DNA packaging into vesicles, that coupled with the inherent technical LoD of the ddPCR platform. The assay successfully identified one APA case that was missed by standard tissue genotyping, highlighting its potential to capture tumor heterogeneity that may be absent in a single tissue biopsy. All 15 IHA plasma samples tested negative for the hotspot mutations, reinforcing the specificity of the assay for the APA subtype (Fig. 4C).

To biologically validate our approach, we analyzed matched peripheral and AVS samples from a patient with *KCNJ5*-mutant APA. As hypothesized, the mutant allele frequency was markedly higher in sEVs isolated from the left adrenal vein compared to PB, while the right side showed minimal signal (Fig. 4B). This result confirms that adrenal-derived mutant sEVs enter the circulation and that their concentration gradients reflect the side of hypersecretion, firmly grounding our plasma-based detection in tumor biology.

We establish the first sEV-based liquid biopsy workflow for endocrine hypertension subtyping, which bridges a critical gap in PA management clinically. It offers a noninvasive alternative to AVS, a technically demanding procedure with 10% failure rates and risks of adrenal hemorrhage, while outperforming imaging modalities like CT/magnetic resonance imaging in specificity (96.7 versus 50 to 60%) (*40*, *41*). By detecting *KCNJ5* mutations present in 43 to 80% of APA cases, the assay directly informs surgical candidacy, potentially reducing unnecessary AVS referrals by 30 to 40%, lowering health care costs and procedural morbidity. The streamlined workflow (under 4 hours) and minimal plasma input (1 ml) further enhance feasibility in resource-limited settings, where AVS expertise is scarce. This accessibility could accelerate early diagnosis for millions of undiagnosed patients with PA, enabling curative adrenalectomy before irreversible cardiovascular damage occurs.

Despite these advances, limitations warrant consideration. First, our immunoaffinity capture relies on antibodies targeting the tetraspanins CD9 and CD63. While this strategy efficiently enriches for a major population of sEVs relevant to our diagnostic purpose, it may introduce a capture bias by potentially under-representing EV subpopulations that lack or express low levels of these specific markers. The modular nature of the HGMS-3D chip platform is well suited to address this in the future by incorporating a broader panel of antibodies (e.g., against CD81 or tissue-specific antigens) to achieve a more holistic EV capture (*42*). The observed variability in sensitivity across mutation subtypes (G151R c.451G>C: 58.33%; G151R c.451G>A: 70.83%; L168R: 69.57%) necessitates larger multicenter validations to clarify biological versus technical contributors. Throughput remains constrained by manual chip operation and RNA extraction steps; future integration with automated microfluidics and direct on-chip lysis could improve scalability. In addition, expanding the mutation panel to include rarer APA drivers would broaden clinical utility. Future iterations could expand coverage by incorporating multiplexed probes for parallel screening of additional PA-related mutations (e.g., CACNA1D and ATP1A1).

It is also important to note the context of our patient cohort. While AVS is the gold standard for subtyping, its invasive nature and technical demands make it difficult to perform routinely, leading to the well-documented underutilization in clinical practice. Therefore, our study enrolled patients with APA based on a combination of conclusive preoperative CT imaging and postoperative pathological/clinical confirmation, a validated diagnostic pathway that does not universally mandate AVS. This approach reflects a common real-world scenario. We successfully analyzed a single AVS sample from a patient with *KCNJ5*-mutant APA, which confirmed the presence of the identical mutation in AVS-derived sEVs, consistent with our peripheral plasma detection. Although this single case lacks statistical power due to the extreme difficulty in procuring such samples, it provides a crucial proof of concept and reinforces the validity of our PB-based assay. The primary goal of our work was to establish a robust noninvasive alternative, and future studies may further explore AVS-derived sEVs as a niche but informative biospecimen.

Our noninvasive sEV-based *KCNJ5* mutation assay does not provide lateralizing information and thus is not a replacement for AVS. Instead, it introduces a complementary, genetic dimension to the diagnostic workflow. It holds promise for application in specific clinical scenarios. For instance, in a patient with a radiologically evident unilateral adrenal nodule, a positive *KCNJ5* mutation test in plasma sEVs could provide strong corroborative genetic evidence for APA. In such cases, particularly where AVS is unavailable, unsuccessful, or declined by the patient, this combined radiological and genetic evidence may suffice to recommend curative adrenalectomy. For patients with atypical or borderline biochemical phenotypes, a positive sEV-based mutation test can serve as a robust qualitative indicator for PA and its surgical subtype, aiding in diagnosis and therapeutic triage. Beyond preoperative diagnosis, this liquid biopsy approach holds promise for postoperative monitoring. For patients with a known preoperative *KCNJ5* mutation, quantitative tracking of the mutant allele frequency in sEVs after adrenalectomy could provide a dynamic, molecular metric to complement the standard clinical and biochemical (PASO) criteria for evaluating surgical success.

Our study demonstrates that the detection of *KCNJ5* mutations in plasma sEVs exhibits high specificity for APA against a background of IHA (all 15 IHA cases tested negative). This supports its utility as a rule-in test for the unilateral, surgical subtype. Looking forward, we envisage a precision medicine approach for PA subtyping. The integration of liquid biopsy (e.g., sEV mutation profiling), targeted metabolomics, and radiomics with clinical indices could enable effective risk stratification. This multimodal strategy could potentially allow a substantial proportion of patients with clear genetic and radiological evidence of APA to proceed directly to curative surgery, thereby reserving the invaluable resource of AVS for diagnostically complex cases where noninvasive tests are inconclusive.

In conclusion, by integrating a high-performance sEV isolation platform with ultrasensitive mutation detection, we have established a liquid biopsy workflow for PA. This approach provides a genetic dimension to subtyping, with the potential to streamline the diagnostic journey for a significant subset of patients. Looking forward, the integration of such molecular tools with imaging and clinical data paves the way for a more precise, stratified management strategy for PA. In the future, the modularity of this platform invites adaptation beyond PA. Its high-efficiency sEV isolation and ultrasensitive detection framework could be extended to other mutation-driven disorders, such as endocrine tumors or hypertension-associated malignancies. Engineering efforts should prioritize multiplexing capacity, workflow automation, and validation across diverse biological matrices to cement its role in precision medicine. By transforming invasive subtyping into a blood-based test, this technology heralds an unprecedented era in the management of endocrine hypertension.

## MATERIALS AND METHODS

### Patient cohort

Patients with a confirmed diagnosis of PA were recruited from the Department of Endocrinology and Hypertension (*43*, *44*). Ethical approval for the study was obtained from the review boards of the Peking Union Medical College Hospital, Chinese Academy of Medical Sciences (I-22PJ964). All patients provided written informed consent prior to enrollment, following a thorough explanation of the study objectives, procedures, and potential implications. The consent covered the collection, processing, storage, and subsequent molecular analysis of plasma and tissue samples for research purposes, including EV isolation and genetic mutation detection. The diagnosis of PA was based on accepted guidelines, including an elevated ARR and confirmatory testing (e.g., saline infusion test or captopril challenge test) where indicated. For inclusion in the APA group, patients were required to have (i) preoperative CT imaging showing a unilateral adrenal nodule, (ii) underwent unilateral adrenalectomy, and (iii) achieved complete biochemical success postoperatively according to the PASO criteria. One exceptional patient, who presented with bilateral adrenal nodules on CT but achieved complete biochemical cure after unilateral adrenalectomy, was also included in the APA group based on the definitive postoperative outcome. Patients failing to achieve complete biochemical cure postoperatively were classified as IHA with a unilateral adrenal nodule.

### Patient samples and plasma processing

PB samples (10 ml) from patients were collected in EDTA-K_2_ anticoagulant tubes. Plasma was separated by centrifugation at 2000*g* for 10 min at room temperature to remove cells and platelets. The resulting platelet-poor plasma was immediately aliquoted and stored at −80°C until further use. For all sEV isolation procedures described in this study, a fixed volume of 1 ml of thawed plasma was used as the input material.

### HGMS-3D chip design and production

The HGMS-3D chip was designed and fabricated using a cylindrical body made of poly(methyl methacrylate) (PMMA), two PMMA external threaded nuts connected by silicone tubes, and a multilayer 400-mesh nickel screen. The cylindrical body has an outer diameter of 1.5 cm, an inner diameter of 1 cm, and a height of 1.5 cm, with internal threads for sealing with the nuts. The opposite ends of the nuts were connected to a silicone tube with an inner diameter of 1.6 mm.

Superparamagnetic beads (1 μm diameter, 10 mg/ml) covalently coupled with a monolayer of streptavidin (100 μl) were combined with 900 μl of phosphate-buffered saline (PBS) in a 1.5-ml centrifuge tube and vortexed for 1 min to mix thoroughly. The mixture was then quickly placed on a magnetic rack for 30 s, the supernatant was removed, and the beads were washed once with PBS. After magnetic separation, 996 μl of PBS was added, followed by vortexing for 1 min. Subsequently, biotinylated anti-CD9 or anti-CD63 antibody (4 μl, 1 mg/ml) was added, mixed, and incubated at room temperature on a horizontal shaker at 1000 rpm for 30 min. A bovine serum albumin (BSA)–PBS solution (80 mg/ml) was prepared as a stock solution, and PBS was diluted with 0.1% BSA solution. After magnetic separation and supernatant removal, 1 ml of 0.1% BSA was added to the tube and vortexed for 1 min. The washing step was repeated four times. Last, the immunomagnetic beads were stored in 1 ml of 0.1% BSA solution at 4°C until use.

Using a syringe and a peristaltic pump, the immunomagnetic beads were injected into the assembled 3D chip, which was then placed on a magnet, thus forming the HGMS-3D chip. All materials were sterilized before use. The 3D chip is reusable and was ultrasonically cleaned with sterile water for three to four cycles. The nickel mesh was ultrasonically cleaned with ethanol while held with tweezers. The silicone tubes were rinsed with 10 ml of sterile water, preferably disposable tubes. All cleaned components were dried in an oven at 60°C.

### Establishment of sEVs isolated by HGMS-3D chip

The HGMS-3D chip was assembled, and immunomagnetic beads conjugated with anti-CD9 and anti-CD63 were injected at a controlled flow rate using a syringe pump. The magnetic beads were uniformly distributed within the 3D chip and promptly placed on a custom apparatus (permanent magnet length: 0.5 cm, yoke thickness: 1.5 cm, and yoke length: 2 cm). Under the high-gradient magnetic field, the beads were evenly anchored on both sides of the nickel mesh, forming a multilayer immunoaffinity microinterface. A small volume of air was then injected to prepare the device for sample loading.

sEVs from 293T cells and human plasma were isolated by HGMS-3D chip. The effects of immunomagnetic bead concentration (0.1, 0.2, 0.5, 1, and 1.5 mg/ml), number of nickel mesh layers (5, 8, 11, and 15), and flow rate (0.02, 0.05, 0.08, 0.1, and 0.2 ml/min) on capture efficiency were evaluated. The sEV DNA was extracted by direct lysis, and the *KCNJ5* gene was detected by qPCR to calculate the relative yield.

For spike-in recovery experiments, sEVs isolated by UC from 300 ml of cell supernatant were added to 1 ml of plasma at volumes of 100, 20, 10, 5, and 0 μl. Plasma sEVs were then captured using the HGMS-3D chip, which was repeated three times. The *KCNJ5* gene was detected by qPCR, and the recovery efficiency was calculated using a standard curve. The amount of *KCNJ5* DNA detected by qPCR is proportional to the number of sEVs present.

$$\text{Recovery}=\frac{{\text{Number}}_{\mathrm{EV}-\mathrm{Recovery}}}{Number_{\mathrm{EV}-\mathrm{Spiked}}}\times 100\%$$

### EV characterization

The sEV solution (10 μl) was dropped on a copper net, incubated at room temperature for 10 min, and washed with sterile distilled water; excess liquid was removed with filter paper. The grid was negatively stained with 10 μl of 2% uranyl acetate for 1 min, blotted dry with filter paper, and air-dried under an incandescent lamp for 2 min. The copper mesh was observed under TEM and imaged at 80 kV. Images were acquired by TEM (TEM-1400plus).

For fluorescence labeling, sEVs were stained with PKH26 red fluorescent dye (Umibio, catalog no. UR52302). The dye was diluted with Diluent C to a working concentration of 100 μM in the dark. After UC, 10 μl of the working dye solution was added to 100 μl of the sEV solution, mixed thoroughly using a vortex mixer for 1 min, and incubated at room temperature for 10 min. Following incubation, 10 ml of 1× PBS was added to the EV-dye complex and mixed thoroughly. Subsequently, excess dye was removed by re-isolating the sEVs using the HGMS-3D chip. Fluorescence images were obtained using an UltraVIEW VOX spinning-disk microscope (PerkinElmer, USA) using a 20×/0.5 numerical aperture air-immersion lens objective and analyzed with Volocity software.

NTA was performed using a ZetaView instrument (Particle Metrix, Germany). sEV samples were diluted 400-fold with PBS and analyzed according to the manufacturer’s guidelines to determine particle size distribution and concentration.

### Western blotting

Radioimmunoprecipitation assay lysis buffer containing phenylmethylsulfonyl fluoride (1:100) and phosphatase inhibitors was prepared on ice. Freshly isolated sEVs were mixed with the lysis buffer and incubated on ice for at least 30 min. Protein concentration was determined using a BCA assay kit. The sEV lysate was combined with 5× SDS–polyacrylamide gel electrophoresis bromophenol blue loading buffer, vortexed thoroughly to ensure complete mixing, and heated at 95° to 100°C for 10 min before use.

Samples were loaded onto precast polyacrylamide gels and electrophoresed at 80 V for 30 min, followed by 120 V for ~1.5 hours. Proteins were transferred to a polyvinylidene difluoride membrane (activated in methanol for 5 min) at 300 mA for 2 hours or 200 mA for 3 hours in cold transfer buffer. The membrane was blocked with 5% nonfat dry milk in phosphate-buffered saline with Tween 20 (PBST) for 1 hour at room temperature and then incubated with primary antibodies (diluted in PBST containing 3% BSA) overnight at 4°C. After five washes with TBST (5 min each), the membrane was incubated with horseradish peroxidase–conjugated secondary antibodies for 1 hour at room temperature. Following five additional washes, protein bands were visualized using an enhanced chemiluminescence (ECL) detection kit, which utilizes horseradish peroxidase (HRP) to catalyze substrate and generate chemiluminescent signals, and imaged with a chemiluminescence imaging system (Tanon-5200, China).

### *KCNJ5* plasmid

The coding sequence of human *KCNJ5* was obtained from NCBI. The WT, G151R (G>A/C), and L168R sequences were synthesized by Sangon Biotech Co., Ltd. (Shanghai, China). Bam HI (5′) and Xho I (3′) restriction sites were incorporated and subsequently ligated into the pcDNA 3.1 cloning vector, resulting in the constructs pcDNA 3.1 WT/G151R (G>A/C)/L168R. Plasmid extraction was performed using a TIANGEN Fast Plasmid Mini Kit [TIANGEN BIOTECH (BEIJING) Co., Ltd., China]. Following digestion with Bam HI, DNA concentration was quantified using a NanoDrop spectrophotometer (converted to copies per microliter). The plasmid was serially diluted with ddH_2_O and added to the lysis system to prepare a standard curve. The remaining plasmid was aliquoted into cryovials and stored at −80°C.

For lentiviral packaging, the same sequences were cloned into the pEZ-Lv208 expression vector using Bam HI and Xho I sites. Ligation products were transformed into competent cells and plated on LB-ampicillin agar. Single colonies were expanded in LB-ampicillin medium for 12 to 16 hours, and the plasmids were verified by Sanger sequencing. Correct clones were further expanded, and plasmid DNA was extracted and stored at −20°C.

### Packaging of lentiviruses

A vial of 293T cells was retrieved and cultured for 48 hours. After digestion with 0.05% trypsin, the cells were seeded into a culture flask coated with 0.1% aqueous solution gel. The cells were incubated overnight, and the density of the 293T cells was monitored. When the cells reached 60 to 80%, they were transfected. The Polyplus-transfection reagent was used for transfection. Five micrograms of the PSPAX2 plasmid, 3.75 μg of the PMD2G plasmid, and 1.25 μg of the plasmid containing the gene of interest were combined in 500 μl of jetPRIME buffer. After vortexing for 10 s, 20 μl of jetPRIME reagent was added and vortexed for an additional 10 s. The mixture was then allowed to stand at room temperature for 10 min. Subsequently, the mixture was carefully added dropwise to the culture medium of human embryonic kidney–293 cells and incubated at 37°C for 4 to 6 hours under standard conditions.

After incubation for 24 hours, green fluorescent protein (GFP) expression was observed. The supernatant was collected at 48 hours, centrifuged at 1500 rpm for 10 min to remove cell debris, and filtered through a 0.45-μm filter. The filtrate was concentrated using a 15-ml ultrafiltration tube (100 kDa molecular weight cutoff) at 5000 rpm for 30 to 45 min at 4°C. The concentrated lentivirus (400 μl) was aliquoted and stored at −80°C.

293T cells are seeded into a six-well plate, ensuring a cell density of 5 × 10^5^ to 1 × 10^6^ cells per well, with 1 ml of Dulbecco’s modified Eagle’s medium culture medium per well. A total of 50 μl of concentrated lentiviral solution was directly added to each well. The plate was centrifuged at 32°C for 1 hour at 2000*g* and then incubated at 37°C. After 2 days, puromycin (1:10,000) was added for selection through medium change, and GFP expression was assessed after 4 days. Once the GFP-positive cells constituted over 95% of the total cell population, we collected the cells and supernatant sEVs, extracted DNA, amplified the *KCNJ5* gene, and performed first-generation sequencing.

### UC for EV isolation

The cell supernatant or plasma was centrifuged at 10,000*g* for 30 min at 4°C to obtain the supernatant. This supernatant was then transferred to a new 50-ml centrifuge tube and centrifuged at 110,000*g* for 70 min at 4°C. After UC was complete, the supernatant was discarded, and the EVs were resuspended in 100 μl of PBS.

### Immunomagnetic bead method for sEV isolation

The cell supernatant or plasma was centrifuged at 10,000*g* for 30 min at 4°C, and the supernatant was collected. The immunomagnetic beads was added to 10 ml of the supernatant and incubated overnight at 4°C to capture sEVs.

### SBI ExoQuick reagent kit for sEV isolation

Following the instructions provided in the SBI ExoQuick Reagent Kit (System Biosciences, USA) manual, the cell supernatant or plasma was centrifuged at 10,000*g* for 30 min at 4°C to obtain the supernatant. The clarified supernatant was mixed with the appropriate volume of ExoQuick solution and incubated at 4°C for 30 min. The mixture was then centrifuged at 1500*g* for 30 min, and the sEV pellet was collected.

### Lysis and extraction of *KCNJ5* DNA/RNA from sEVs

Following the isolation of sEVs using the HGMS-3D chip, the custom magnet was removed, and the nickel mesh was transferred into a centrifuge tube. Subsequently, 50 μl of PBS and 2 μl of a proteinase K solution (20 mg/ml) were added, and the mixture was incubated at 56°C for 15 min to facilitate digestion. To inactivate proteinase K, samples were heated for 5 min at 95°C using a PCR thermal cycler. The lysate was either used immediately for ddPCR or stored at −80°C.

### qPCR

The 20-μl PCR mixture contained 10 μl of 2× PerfectStart Green qPCR SuperMix, 0.5 μl of *KCNJ5* G151R P-F (10 μM) and *KCNJ5* G151R P-R (10 μM) primer, and 9 μl of extracted DNA. The primer sequences used were as follows: *KCNJ5* G151R P-F, 5′-TATGTCCCCATTGCCAC-3′; *KCNJ5* G151R P-R, 5′-AGGGCTCTTCTGGTTGA-3′. Amplification was performed using the following protocol: 30 s at 95°C and 44 cycles of 5 s at 95°C and 30 s at 60°C. Real-time amplification reactions were performed using a CFX 96 Real-Time System (Bio-Rad). Using qPCR to detect the quantification cycle (*C*q) values of the *KCNJ5* gene in EVs isolated by UC and the HGMS-3D chip device, the relative yield of EVs isolated by the 3D chip device compared with that achieved by UC was calculated using the following formula

$$\text{Relative yield}=2^{\text{C}\text{q UC}-\text{C}\text{q}\;\text{other method}}$$

### Sanger sequencing

For genome extraction from PB and tumor tissues, the instruction manual of the TIANamp Genomic DNA Kit [Tiangen Biotechnology (Beijing) Co., Ltd.] was followed. For *KCNJ5* gene G151R (c.451G>C), G151R (c.451G>A), and L168R (c.503T>G) mutation analyses, Sanger sequencing primers *KCNJ5*(91–858) (forward) 5′-GATTATGTCCCCATTGCCAC-3′ and *KCNJ5* (91–858) (reverse) 5′-GAAAGGGCTCTTCTGGTTGA-3′ were used for the PCR reaction. PCR amplification conditions were as follows: 95°C for 5 min, 95°C for 30 s, 56°C for 30 s, 72°C for 1 min and 36 cycles, and 72°C for 10 min. The PCR products were sent to Sangon Biotech Co., Ltd. (Shanghai, China) for Sanger sequencing.

### Droplet digital PCR

The ddPCR was carried out using the OsciDrop dPCR system, as previously described by Li *et al.* (*38*). Briefly, the dPCR reaction mix in eight-strip PCR tubes was prepared by combining 5 μl of 5× one-step reverse transcription (RT) dPCR, 1.25 μl of Primer and Probe (10 μM/4 μM), and 18.25 μl of template-extracted DNA/RNA from EV samples, resulting in a final volume of 25 μl. Once the samples and consumables were ready, the automated dPCR process was initiated, which began by loading dPCR plates with 8 ml of droplet generation oil using an integrated piston pump. The droplet printer coordinated the four-channel syringe pump using 50-μl glass syringes (30-mm stroke, SETonic GmbH, Ilmenau, Germany) and a customized rotary motor with an oscillating frequency of 122 Hz to generate 20,000 1-nanoliter droplets for each sample. Following droplet generation, the thermal blocks were subjected to in situ PCR thermocycling. The RT-dPCR amplification conditions were as follows: 55°C for 20 min, 95°C for 2 min, 95°C for 30 s, and 56°C for 30 s and 45 cycles. Subsequently, the dPCR system was used for in situ multicolor fluorescence imaging at 58°C. To enable intelligent image analysis using the OsciDrop dPCR system, we integrated supervised deep neural networks for droplet feature extraction and filtering within the software.

### Statistical analysis

Data analysis was performed using GraphPad Prism 10 (GraphPad Software, Inc., San Diego, CA, USA) and MedCalc version 23 (MedCalc Software Ltd., Ostend, Belgium). Three replications were performed for each data point on each graph and are represented as the means ± SD. Differences between groups were assessed using the Student’s *t* test. Statistical significance was set at *P* < 0.05. ROC curves were constructed using the MedCalc software to assess the accuracy of APA diagnosis based on *KCNJ5* mutation detection in EV. All experiments were replicated at least three times independently.

## References

[1] A. F. Turcu, J. Yang, A. Vaidya, Primary aldosteronism—A multidimensional syndrome. Nat. Rev. Endocrinol. 18, 665–682 (2022). 36045149 10.1038/s41574-022-00730-2

[2] M.-C. Zennaro, S. Boulkroun, F. L. Fernandes-Rosa, Pathogenesis and treatment of primary aldosteronism. Nat. Rev. Endocrinol. 16, 578–589 (2020). 32724183 10.1038/s41574-020-0382-4

[3] G. Maiolino, L. A. Calò, G. P. Rossi, The time has come for systematic screening for primary aldosteronism in all hypertensives. J. Am. Coll. Cardiol. 69, 1821–1823 (2017). 28385311 10.1016/j.jacc.2017.02.041

[4] W. F. Young Jr., Diagnosis and treatment of primary aldosteronism: Practical clinical perspectives. J. Intern. Med. 285, 126–148 (2019). 30255616 10.1111/joim.12831

[5] J. W. Funder, Primary aldosteronism and cardiovascular risk, before and after treatment. Lancet Diabetes Endocrinol. 6, 5–7 (2018). 29129574 10.1016/S2213-8587(17)30368-6

[6] E. A. B. Azizan, W. M. Drake, M. J. Brown, Primary aldosteronism: Molecular medicine meets public health. Nat. Rev. Nephrol. 19, 788–806 (2023). 37612380 10.1038/s41581-023-00753-6 PMC7615304

[7] N. Mullen, J. Curneen, P. T. Donlon, P. Prakash, I. Bancos, M. Gurnell, M. C. Dennedy, Treating primary aldosteronism-induced hypertension: Novel approaches and future outlooks. Endocr. Rev. 45, 125–170 (2024). 37556722 10.1210/endrev/bnad026 PMC10765166

[8] M. Reincke, I. Bancos, P. Mulatero, U. I. Scholl, M. Stowasser, T. A. Williams, Diagnosis and treatment of primary aldosteronism. Lancet Diabetes Endocrinol. 9, 876–892 (2021). 34798068 10.1016/S2213-8587(21)00210-2

[9] G. P. Rossi, Primary aldosteronism: JACC state-of-the-art review. J. Am. Coll. Cardiol. 74, 2799–2811 (2019). 31779795 10.1016/j.jacc.2019.09.057

[10] G. P. Rossi, T. M. Seccia, G. Palumbo, A. Belfiore, G. Bernini, G. Caridi, G. Desideri, B. Fabris, C. Ferri, G. Giacchetti, C. Letizia, M. MacCario, F. Mallamaci, M. Mannelli, A. Patalano, D. Rizzoni, E. Rossi, A. C. Pessina, F. Mantero, Within-patient reproducibility of the aldosterone: Renin ratio in primary aldosteronism. Hypertension 55, 83–89 (2010). 19933925 10.1161/HYPERTENSIONAHA.109.139832

[11] G. P. Rossi, M. Barisa, A. Belfiore, G. Desideri, C. Ferri, C. Letizia, M. MacCario, A. Morganti, G. Palumbo, A. Patalano, E. Roman, T. M. Seccia, A. C. Pessina, F. Mantero, The aldosterone-renin ratio based on the plasma renin activity and the direct renin assay for diagnosing aldosterone-producing adenoma. J. Hypertens. 28, 1892–1899 (2010). 20683340 10.1097/HJH.0b013e32833d2192

[12] J. W. Funder, R. M. Carey, F. Mantero, M. H. Murad, M. Reincke, H. Shibata, M. Stowasser, W. F. Young Jr., The management of primary aldosteronism: Case detection, diagnosis, and treatment: An endocrine society clinical practice guideline. J. Clin. Endocrinol. Metab. 101, 1889–1916 (2016). 26934393 10.1210/jc.2015-4061

[13] T. A. Williams, M. Reincke, Pathophysiology and histopathology of primary aldosteronism. Trends Endocrinol. Metab. 33, 36–49 (2022). 34743804 10.1016/j.tem.2021.10.002

[14] T. A. Williams, J. W. M. Lenders, P. Mulatero, J. Burrello, M. Rottenkolber, C. Adolf, F. Satoh, L. Amar, M. Quinkler, J. Deinum, F. Beuschlein, K. K. Kitamoto, U. Pham, R. Morimoto, H. Umakoshi, A. Prejbisz, T. Kocjan, M. Naruse, M. Stowasser, T. Nishikawa, W. F. Young Jr., C. E. Gomez-Sanchez, J. W. Funder, M. Reincke, Primary Aldosteronism Surgery Outcome (PASO) investigators, Outcomes after adrenalectomy for unilateral primary aldosteronism: An international consensus on outcome measures and analysis of remission rates in an international cohort. Lancet Diabetes Endocrinol. 5, 689–699 (2017). 28576687 10.1016/S2213-8587(17)30135-3 PMC5572673

[15] G. P. Rossi, P. Mulatero, F. Satoh, 10 good reasons why adrenal vein sampling is the preferred method for referring primary aldosteronism patients for adrenalectomy. J. Hypertens. 37, 603–611 (2019). 30431526 10.1097/HJH.0000000000001939

[16] A. Vaidya, P. Mulatero, R. Baudrand, G. K. Adler, The expanding spectrum of primary aldosteronism: implications for diagnosis, pathogenesis, and treatment. Endocr. Rev. 39, 1057–1088 (2018). 30124805 10.1210/er.2018-00139 PMC6260247

[17] M. Reincke, Adrenal vein sampling for subtyping in primary aldosteronism. Lancet Diabetes Endocrinol. 4, 718–719 (2016). 27325146 10.1016/S2213-8587(16)30113-9

[18] Y. Yang, C. E. Gomez-Sanchez, D. Jaquin, E. T. Aristizabal Prada, L. S. Meyer, T. Knösel, H. Schneider, F. Beuschlein, M. Reincke, T. A. Williams, Primary Aldosteronism: KCNJ5 mutations and adrenocortical cell growth. Hypertension 74, 809–816 (2019). 31446799 10.1161/HYPERTENSIONAHA.119.13476 PMC6739168

[19] U. I. Scholl, Genetics of primary aldosteronism. Hypertension 79, 887–897 (2022). 35139664 10.1161/HYPERTENSIONAHA.121.16498 PMC8997684

[20] S. S. Park, C. H. Ahn, S. W. Kim, J.-M. Koh, S. H. Lee, J. H. Kim, Temporal trends in clinical features of patients with primary aldosteronism over 20 years. Hypertens. Res. 47, 2019–2028 (2024). 38760522 10.1038/s41440-024-01703-w PMC11298405

[21] S. Das, C. J. Lyon, T. Hu, A panorama of extracellular vesicle applications: From biomarker detection to therapeutics. ACS Nano 18, 9784–9797 (2024). 38471757 10.1021/acsnano.4c00666 PMC11008359

[22] C.-C. Hsu, Y. Yang, E. Kannisto, X. Zeng, G. Yu, S. K. Patnaik, G. K. Dy, M. E. Reid, Q. Gan, Y. Wu, Simultaneous detection of tumor derived exosomal protein–microRNA pairs with an Exo-PROS biosensor for cancer diagnosis. ACS Nano 17, 8108–8122 (2023). 37129374 10.1021/acsnano.2c10970 PMC10266547

[23] C. Park, S. Chung, H. Kim, N. Kim, H. Y. Son, R. Kim, S. Lee, G. Park, H. W. Rho, M. Park, J. Han, Y. Song, J. Lee, S.-H. Jun, Y.-M. Huh, H. H. Jeong, E.-K. Lim, E. Kim, S. Haam, All-in-one fusogenic nanoreactor for the rapid detection of exosomal microRNAs for breast cancer diagnosis. ACS Nano 18, 26297–26314 (2024). 10.1021/acsnano.4c08339 39248519

[24] J. Song, M. H. Cho, H. Cho, Y. Song, S. W. Lee, H. C. Nam, T. H. Yoon, J. C. Shin, J.-S. Hong, Y. Kim, E. Ekanayake, J. Jeon, D. G. You, S. G. Im, G.-S. Choi, J. S. Park, B. C. Carter, L. Balaj, A. N. Seo, M. A. Miller, S. Y. Park, T. Kang, C. M. Castro, H. Lee, Amplifying mutational profiling of extracellular vesicle mRNA with SCOPE. Nat. Biotechnol. 43, 1485–1495 (2025). 39375445 10.1038/s41587-024-02426-6 PMC12747296

[25] C. Kahlert, Liquid biopsy: Is there an advantage to analyzing circulating exosomal DNA compared to cfDNA or are they the same? Cancer Res. 79, 2462–2465 (2019). 31043377 10.1158/0008-5472.CAN-19-0019

[26] W. Yu, J. Hurley, D. Roberts, S. K. Chakrabortty, D. Enderle, M. Noerholm, X. O. Breakefield, J. K. Skog, Exosome-based liquid biopsies in cancer: Opportunities and challenges. Ann. Oncol. 32, 466–477 (2021). 33548389 10.1016/j.annonc.2021.01.074 PMC8268076

[27] E. H. Koritzinsky, J. M. Street, R. A. Star, P. S. T. Yuen, Quantification of exosomes. J. Cell. Physiol. 232, 1587–1590 (2017). 27018079 10.1002/jcp.25387 PMC5039048

[28] S. Li, L. Zhu, L. Zhu, X. Mei, W. Xu, A sandwich-based evanescent wave fluorescent biosensor for simple, real-time exosome detection. Biosens. Bioelectron. 200, 113902 (2022). 34954570 10.1016/j.bios.2021.113902

[29] L. Zhu, H.-T. Sun, S. Wang, S.-L. Huang, Y. Zheng, C.-Q. Wang, B.-Y. Hu, W. Qin, T.-T. Zou, Y. Fu, X.-T. Shen, W.-W. Zhu, Y. Geng, L. Lu, H.-L. Jia, L.-X. Qin, Q.-Z. Dong, Isolation and characterization of exosomes for cancer research. J. Hematol. Oncol. 13, 152 (2020). 33168028 10.1186/s13045-020-00987-y PMC7652679

[30] R. Chinnappan, Q. Ramadan, M. Zourob, An integrated lab-on-a-chip platform for pre-concentration and detection of colorectal cancer exosomes using anti-CD63 aptamer as a recognition element. Biosens. Bioelectron. 220, 114856 (2023). 36395728 10.1016/j.bios.2022.114856

[31] S. Marczak, K. Richards, Z. Ramshani, E. Smith, S. Senapati, R. Hill, D. B. Go, H.-C. Chang, Simultaneous isolation and preconcentration of exosomes by ion concentration polarization. Electrophoresis 39, 2029–2038 (2018). 10.1002/elps.201700491 PMC6110980 29484678

[32] S.-L. Sim, T. He, A. Tscheliessnig, M. Mueller, R. B. H. Tan, A. Jungbauer, Protein precipitation by polyethylene glycol: A generalized model based on hydrodynamic radius. J. Biotechnol. 157, 315–319 (2012). 22001847 10.1016/j.jbiotec.2011.09.028

[33] H. Cui, T. Zheng, N. Qian, X. Fu, A. Li, S. Xing, X.-F. Wang, Aptamer-functionalized magnetic Ti_3_C_2_ based nanoplatform for simultaneous enrichment and detection of exosomes. Small 20, e2402434 (2024). 38970554 10.1002/smll.202402434

[34] K. Yi, Y. Wang, Y. Rong, Y. Bao, Y. Liang, Y. Chen, F. Liu, S. Zhang, Y. He, W. Liu, C. Zhu, L. Wu, J. Peng, H. Chen, W. Huang, Y. Yuan, M. Xie, F. Wang, Transcriptomic signature of 3D hierarchical porous chip enriched exosomes for early detection and progression monitoring of hepatocellular carcinoma. Adv. Sci. 11, e2305204 (2024). 10.1002/advs.202305204 PMC11005692 38327127

[35] M. Wu, Y. Ouyang, Z. Wang, R. Zhang, P.-H. Huang, C. Chen, H. Li, P. Li, D. Quinn, M. Dao, S. Suresh, Y. Sadovsky, T. J. Huang, Isolation of exosomes from whole blood by integrating acoustics and microfluidics. Proc. Natl. Acad. Sci. U.S.A. 114, 10584–10589 (2017). 28923936 10.1073/pnas.1709210114 PMC5635903

[36] K. Zhang, Y. Yue, S. Wu, W. Liu, J. Shi, Z. Zhang, Rapid capture and nondestructive release of extracellular vesicles using aptamer-based magnetic isolation. ACS Sens. 4, 1245–1251 (2019). 30915846 10.1021/acssensors.9b00060

[37] S. Monticone, J. Burrello, D. Tizzani, C. Bertello, A. Viola, F. Buffolo, L. Gabetti, G. Mengozzi, T. A. Williams, F. Rabbia, F. Veglio, P. Mulatero, Prevalence and clinical manifestations of primary aldosteronism encountered in primary care practice. J. Am. Coll. Cardiol. 69, 1811–1820 (2017). 28385310 10.1016/j.jacc.2017.01.052

[38] C. Li, N. Kang, S. Ye, W. Huang, X. Wang, C. Wang, Y. Li, Y.-F. Liu, Y. Lan, L. Ma, Y. Zhao, Y. Han, J. Fu, D. Shen, L. Dong, W. Du, All-in-one OsciDrop digital PCR system for automated and highly multiplexed molecular diagnostics. Adv. Sci. 11, e2309557 (2024). 10.1002/advs.202309557 PMC11151056 38516754

[39] S. C. Taylor, G. Laperriere, H. Germain, Droplet digital PCR versus qPCR for gene expression analysis with low abundant targets: From variable nonsense to publication quality data. Sci. Rep. 7, 2409 (2017). 28546538 10.1038/s41598-017-02217-x PMC5445070

[40] G. P. Rossi, R. J. Auchus, M. Brown, J. W. M. Lenders, M. Naruse, P. F. Plouin, F. Satoh, W. F. Young, An expert consensus statement on use of adrenal vein sampling for the subtyping of primary aldosteronism. Hypertension 63, 151–160 (2014). 24218436 10.1161/HYPERTENSIONAHA.113.02097

[41] T. Kocjan, M. Jensterle, G. Vidmar, R. Vrckovnik, P. Berden, M. Stankovic, Adrenal vein sampling for primary aldosteronism: A 15-year national referral center experience. Radiol. Oncol. 54, 409–418 (2020). 32889797 10.2478/raon-2020-0052 PMC7585337

[42] V. Barreca, Z. Boussadia, D. Polignano, L. Galli, V. Tirelli, M. Sanchez, M. Falchi, L. Bertuccini, F. Iosi, M. Tatti, M. Sargiacomo, M. L. Fiani, Metabolic labelling of a subpopulation of small extracellular vesicles using a fluorescent palmitic acid analogue. J. Extracell. Vesicles 12, e12392 (2023). 38072803 10.1002/jev2.12392 PMC10710952

[43] W. F. Young Jr., Pathophysiology and clinical features of primary aldosteronism (2024); www.uptodate.cn/contents/pathophysiology-and-clinical-features-of-primary-aldosteronism/print?sectionName=.

[44] W. F. Young Jr., Diagnosis of primary aldosteronism (2025); www.uptodate.cn/contents/diagnosis-of-primary-aldosteronism/print?search=.

