# Supplementary material for: High-gradient magnetic separation chip–based small extracellular vesicle isolation for noninvasive subtyping of primary aldosteronism
Source: Sci Adv. 2026 Apr 17;12(16):eaeb4949. doi: 10.1126/sciadv.aeb4949 (PMC13089352; doi:10.1126/sciadv.aeb4949)
Supplement: Supplementary file 1 — Supplementary Materials and Methods Tables S1 to S3 Fig. S1 [file sciadv.aeb4949_sm.pdf]

Science Advances
AAAS

Supplementary Materials for

High-gradient magnetic separation chip–based small extracellular vesicle isolation for noninvasive subtyping of primary aldosteronism

Dong Wang *et al.*

Corresponding author: Yushi Zhang, beijingzhangyushi@126.com; Mingzhu Yang, yixin051@163.com

Sci. Adv. **12**, eaeb4949 (2026)
DOI: 10.1126/sciadv.aeb4949

This PDF file includes:

Supplementary Materials and Methods
Tables S1 to S3
Fig. S1

MATERIALS AND METHODS

Materials and reagents

Biotinylated Anti-CD63 antibody (ab134331, Abcam, USA), biotinylated Anti-CD9 antibody (ab28094, Abcam, USA), Dynabeads™ MyOne™ Streptavidin C1 magnetic beads (65002, Thermo Fisher, USA), 400 mesh nickel screen (Aodeyuan Metal Products Co., Ltd., China), 1.6 mm inner diameter silicone tubing (14#, Baoding Lange, China), 2 mL and 10 mL syringes (Zhiyu, China), PBS solution (SH30256, Hyclone, USA), BSA (Sigma, USA), Exoquick reagent kit (EXOQ5A-1, SBI, China), exosome inhibitor GW4869 (HY-19363, MCE, USA), exosome red fluorescent labeling dye (PKH26) (UR52302, Umibio, USA).TGS101 antibody (67381-1-lg, Proteintech, USA), CD63 antibody (25682-1-AP, Proteintech, USA), Calnexin antibody (10427-2-AP, Proteintech, USA), HSP 70 antibody (4872, CST, USA), Alix antibody (ab275377, Abcam, USA), RIPA lysis buffer (P0013B, Beyotime Bio, China), PMSF (P0100, Solarbio, China), 5×SDS-PAGE bromophenol blue loading buffer (20315ES05, YEASEN, China), protein marker (26619, Thermo Pierce, USA), BCA protein

concentration detection kit (P0012, Beyotime Bio, China), methanol (80080418, Sinopharm, China), electrophoresis buffer (NuPAGE Running Buffer 20X, Thermo, USA), transfer buffer (NuPAGE Transfer Buffer 20X, Thermo, USA), non-fat dry milk (232100, BD, USA), detection reagent (Immobilon Western Chemiluminescent HRP, USA), TWEEN 20 (Sigma, USA), PVDF membrane (Immobilon-P Transfer Membranes, USA).EDTA tubes (367861, BD Vacutainer, USA), RNAlater solution (AM7024, Thermo, USA), DNA extraction kit (TIANamp Genomic DNA Kit, USA), Proteinase K (Sangon Biotech Co., Ltd., China), Premix Ex Taq (Probe qPCR) (RR390A, TAKARA, Dalian), OsciDrop dPCR consumables, reagents, and 5X one-step RT-dPCR MIX kits (Maccura Biotech, China).

Table S1. Primer probes for KCNJ5 mutation

**Genotype**	**Sequence**
Forword	CCTGTTCTCCATTGAGACCGA
Reverse	TGACGATGGAGCCCAGGAT
G151R（c.451 G>A）prob	FAM-CATT/LNA_A/GGTATGG/LNA_C/TTCCG-BHQ1
G151R（c.451 G>C）prob	ROX-/LNA_C/ATT/LNA_C/GGTATGGCTTCC-BHQ2
L168R（c. 503T>G）prob	HEX-TGGACCA/LNA_A/G/LNA_C/GGAGTATAA-BHQ1
Wild Type	CY5- CTGGAC/LNA_C/AAG/LNA_A/GGAGTATAATC - BHQ3

Table.S2 The MAF (%) of KCNJ5 mutation in sEVs

No.	Clinical Diagnosis	KCNJ5 status in tissue	G151R(c.451G>A) MAF(%) in sEVs	G151R(c.451G>C) MAF(%) in sEVs	L168R(c.503T>G) MAF(%) in sEVs
1	APA	G151R(c.451G>A)	3.29%	0.00%	0.00%
2	APA	G151R(c.451G>C)	0.00%	0.00%	0.03%
3	APA	Wild	0.00%	0.00%	0.00%
4	APA	Wild	0.00%	0.00%	0.00%
5	APA	L168R(c.503T>G)	0.00%	0.00%	9.33%
6	APA	G151R(c.451G>A)	4.22%	0.03%	0.00%
7	APA	G151R(c.451G>C)	0.00%	0.00%	0.00%
8	APA	Wild	0.00%	0.00%	0.00%
9	APA	Wild	0.00%	0.00%	0.00%
10	APA	Wild	0.00%	0.00%	0.00%
11	APA	G151R(c.451G>A)	2.99%	0.00%	0.00%
12	APA	Wild	0.00%	0.00%	0.00%

13	APA	Wild	0.00%	0.00%	0.00%
14	APA	Wild	0.00%	0.00%	0.00%
15	APA	Wild	0.00%	0.00%	0.00%
16	APA	G151R(c.451G>C)	0.00%	2.72%	0.00%
17	APA	G151R(c.451G>A)	1.05%	0.00%	0.00%
18	APA	G151R(c.451G>A)	1.71%	0.00%	0.08%
19	APA	L168R(c.503T>G）	0.00%	0.00%	2.62%
20	APA	G151R(c.451G>A)	1.27%	0.00%	0.00%
21	APA	Wild	0.00%	0.00%	0.00%
22	APA	L168R(c.503T>G）	0.00%	0.00%	4.13%
23	APA	G151R(c.451G>A)	1.22%	0.00%	0.00%
24	APA	Wild	0.00%	0.00%	0.00%
25	APA	L168R(c.503T>G）	0.00%	0.00%	0.90%
26	APA	L168R(c.503T>G）	0.00%	0.00%	1.94%
27	APA	L168R(c.503T>G）	0.00%	0.00%	3.00%
28	APA	G151R(c.451G>A)	1.04%	0.00%	0.00%
29	APA	Wild	0.00%	0.02%	0.00%
30	APA	Wild	0.00%	0.00%	0.00%
31	APA	Wild	0.02%	0.00%	0.00%
32	APA	G151R(c.451G>A)	0.00%	0.00%	0.00%
33	APA	L168R(c.503T>G）	0.00%	0.00%	1.79%
34	APA	L168R(c.503T>G）	0.00%	0.00%	1.18%
35	APA	G151R(c.451G>A)	0.47%	0.02%	0.04%
36	APA	L168R(c.503T>G）	0.04%	0.00%	0.00%
37	APA	G151R(c.451G>A)	0.99%	0.03%	0.00%
38	APA	Wild	0.05%	0.05%	0.00%
39	APA	Wild	0.07%	0.00%	0.00%
40	APA	G151R(c.451G>A)	0.34%	0.00%	0.00%
41	APA	Wild	0.00%	0.00%	0.00%
42	APA	G151R(c.451G>A)	0.86%	0.00%	0.00%
43	APA	G151R(c.451G>A)	0.96%	0.00%	0.00%
44	APA	G151R(c.451G>C)	0.00%	2.11%	0.00%
45	APA	G151R(c.451G>A)	0.18%	0.03%	0.03%
46	APA	G151R(c.451G>C)	0.04%	2.85%	0.00%
47	APA	G151R(c.451G>A)	1.10%	0.00%	0.00%
48	APA	L168R(c.503T>G）	0.00%	0.00%	0.00%
49	APA	L168R(c.503T>G）	0.00%	0.00%	1.46%
50	APA	G151R(c.451G>C)	0.03%	2.42%	0.00%
51	APA	G151R(c.451G>C)	0.03%	1.87%	0.00%
52	APA	L168R(c.503T>G）	0.00%	0.00%	0.00%
53	APA	Wild	0.02%	0.00%	4.44%
54	APA	G151R(c.451G>C)	0.00%	0.00%	0.03%
55	APA	G151R(c.451G>A)	1.72%	0.00%	0.00%
56	APA	Wild	0.00%	0.00%	0.00%

57	APA	Wild	0.00%	0.00%	0.00%
58	APA	Wild	0.00%	0.00%	0.00%
59	APA	Wild	0.00%	0.00%	0.00%
60	APA	Wild	0.00%	0.00%	0.00%
61	APA	Wild	0.03%	0.00%	0.00%
62	APA	Wild	0.00%	0.00%	0.00%
63	APA	Wild	0.00%	0.00%	0.00%
64	APA	G151R(c.451G>A)	0.00%	0.02%	0.00%
65	APA	G151R(c.451G>A)	0.00%	0.00%	0.02%
66	APA	Wild	0.00%	0.00%	0.00%
67	APA	L168R(c.503T>G）	0.00%	0.00%	0.00%
68	APA	Wild	0.00%	0.00%	0.00%
69	APA	Wild	0.00%	0.00%	0.00%
70	APA	Wild	0.00%	0.00%	0.00%
71	APA	Wild	0.00%	0.00%	0.00%
72	APA	L168R(c.503T>G）	0.00%	0.02%	0.12%
73	APA	G151R(c.451G>C)	0.00%	0.00%	0.00%
74	APA	Wild	0.02%	0.00%	0.00%
75	APA	G151R(c.451G>A)	0.00%	0.00%	0.00%
76	APA	G151R(c.451G>A)	0.00%	0.00%	0.00%
77	APA	G151R(c.451G>C)	0.00%	0.16%	0.00%
78	APA	Wild	0.00%	0.02%	0.05%
79	APA	L168R(c.503T>G）	0.00%	0.00%	0.33%
80	APA	Wild	0.00%	0.09%	0.00%
81	APA	Wild	0.00%	0.00%	0.00%
82	APA	Wild	0.00%	0.00%	0.00%
83	APA	L168R(c.503T>G）	0.00%	0.00%	1.65%
84	APA	Wild	0.00%	0.07%	0.00%
85	APA	Wild	0.00%	0.00%	0.00%
86	APA	Wild	0.00%	0.00%	0.00%
87	APA	L168R(c.503T>G）	0.00%	0.00%	0.00%
88	APA	L168R(c.503T>G）	0.00%	0.00%	0.00%
89	APA	Wild	0.00%	0.00%	0.00%
90	APA	Wild	0.01%	0.00%	0.00%
91	APA	L168R(c.503T>G）	0.00%	0.00%	0.86%
92	APA	Wild	0.00%	0.00%	0.00%
93	APA	L168R(c.503T>G）	0.00%	0.00%	1.05%
94	APA	Wild	0.00%	0.00%	0.00%
95	APA	Wild	0.01%	0.00%	0.00%
96	APA	G151R(c.451G>C)	0.00%	0.00%	0.00%
97	APA	G151R(c.451G>A)	0.00%	0.00%	0.00%
98	APA	L168R(c.503T>G）	0.00%	0.00%	0.00%
99	APA	G151R(c.451G>A)	0.02%	0.00%	0.00%
100	APA	G151R(c.451G>C)	0.00%	1.30%	0.00%

101	APA	Wild	0.00%	0.00%	0.00%
102	APA	Wild	0.00%	0.00%	0.00%
103	APA	L168R(c.503T>G）	0.00%	0.00%	0.00%
104	APA	L168R(c.503T>G）	0.00%	0.00%	2.65%
105	APA	Wild	0.00%	0.00%	0.00%
106	APA	G151R(c.451G>A)	0.25%	0.02%	0.00%
106-R	APA	G151R(c.451G>A)	0.12%	-	-
106-L	APA	G151R(c.451G>A)	0.55%	-	-
107	IHA	Wild	0.00%	0.00%	0.00%
108	IHA	Wild	0.00%	0.00%	0.02%
109	IHA	Wild	0.00%	0.00%	0.03%
110	IHA	Wild	0.02%	0.02%	0.00%
111	IHA	Wild	0.01%	0.01%	0.03%
112	IHA	Wild	0.04%	0.00%	0.02%
113	IHA	Wild	0.00%	0.00%	0.00%
114	IHA	Wild	0.05%	0.03%	0.00%
115	IHA	Wild	0.03%	0.03%	0.00%
116	IHA	Wild	0.00%	0.00%	0.00%
117	IHA	Wild	0.00%	0.00%	0.02%
118	IHA	Wild	0.00%	0.00%	0.00%
119	IHA	Wild	0.00%	0.00%	0.00%
120	IHA	Wild	0.02%	0.00%	0.00%
121	IHA	Wild	0.00%	0.00%	0.00%
122	Non-PA	Wild	0.00%	0.00%	0.00%
123	Non-PA	Wild	0.00%	0.01%	0.00%
124	Non-PA	Wild	0.02%	0.00%	0.00%
125	Non-PA	Wild	0.02%	0.03%	0.00%
126	Non-PA	Wild	0.00%	0.00%	0.04%
127	Non-PA	Wild	0.00%	0.00%	0.00%
128	Non-PA	Wild	0.00%	0.00%	0.04%
129	Non-PA	Wild	0.00%	0.00%	0.00%

Table.S3 The information of clinical samples

Patient			Clinical and Biochemical Characteristics											Clinical and Biochemical Characteristics After operation							
No.	Age at diagnosis, y	Gender	Duration of hypertension, y	SBP, mmHg	DBP, mmHg	Lowest K, mmol/L	PAC, ng.dL-1	PRA, ng/mL/h	CT (APA-1, IHA-2)	CT (size of largest nodule, mm)	KCNJ5 mutation (No-0, Yes-1)	KCNJ5 mutation (WT, G151R, L168R)	Clinical Diagnosis (APA, IHA)	SBP, mmHg	DBP, mmHg	Lowest K, mmol/L	PAC, ng.dL-1	PRA, ng/mL/h	ARR, ng.dL–1/ng.mL–1.h–1	Clinical success (Absent-0, Partial-1, Complete-2)	Biochemical success (Absent-0, Partial-1, Complete-2)
1	50	F	20	160	100	3.8	25.7	0.01	1	20	1	G151R	APA	108	62	4.3	10.5	0.61	17.2	2	2
2	49	M	15	198	133	3.1	17.9	0.01	1	21	1	G151R	APA	131	76	4.2	8.8	0.36	24.4	2	2
3	64	F	27	180	110	3.2	20.2	0.01	1	10	0	WT	APA	112	65	4.6	7.1	1.26	5.6	2	2
4	61	F	30	185	100	3.2	16.9	0.01	1	15	0	WT	APA	120	80	4	6.5	0.72	9	2	2
5	32	F	1	177	125	2.3	28.7	0.225	1	23	1	L168R	APA	122	74	4.5	7	0.34	20.5	2	2
6	46	F	16	180	105	3	57.6	0.01	1	18	1	G151R	APA	114	63	4.9	6.02	0.26	23.2	2	2
7	42	F	13	180	120	3.4	17.8	0.01	1	16	1	G151R	APA	129	80	4.3	12.6	0.61	20.7	2	2
8	49	F	10	185	106	4	16.2	0.33	1	11	0	WT	APA	104	78	4.3	9.3	2.83	3.3	2	2
9	45	F	8	180	110	3.1	33.3	0.12	1	20	0	WT	APA	113	71	4	9.2	0.62	14.8	2	2
10	44	F	14	202	122	3	17.9	0.11	1	15	0	WT	APA	111	70	4.2	6.8	1.13	6	2	2
11	38	M	6	189	126	2.8	20.3	0.01	1	18	1	G151R	APA	122	72	4.4	9.1	0.37	24.6	2	2
12	60	F	23	203	115	3.6	19.2	0.01	1	8	0	WT	APA	145	92	4.2	6	0.49	12.2	1	2
13	65	M	35	157	95	3.7	20.7	0.01	1	17	0	WT	APA	129	76	4.9	7.5	0.82	9.1	2	2
14	66	F	13	166	110	3.5	22.1	0.01	1	15	0	WT	APA	105	62	4.4	12.9	1.35	9.5	2	2
15	50	F	19	190	120	3.2	16.9	0.31	1	15	0	WT	APA	125	71	4.2	10.2	0.99	10.3	2	2
16	50	F	17	185	105	3.7	22.6	0.01	1	15	1	G151R	APA	120	83	5.1	8.1	1.39	5.8	2	2
17	36	F	8	190	106	3.8	17.3	0.01	1	22	1	G151R	APA	129	77	4.8	11	0.48	22.9	2	2
18	64	F	14	210	130	3.4	22.1	0.01	1	14	1	G151R	APA	112	78	4.2	7.5	2.63	2.9	2	2
19	62	F	30	160	110	2.6	17.1	0.01	1	26	1	L168R	APA	108	81	4.1	5.4	0.27	20	2	2
20	47	F	17	170	110	2.7	20.2	0.01	1	23	1	G151R	APA	139	88	4.4	6.5	0.63	10.3	1	2
21	60	F	29	160	101	3.2	32.2	0.01	1	10	0	WT	APA	121	63	4.3	7.6	0.89	8.5	2	2
22	34	M	3	165	100	2.9	30.1	0.01	1	13	1	L168R	APA	127	67	4.5	11.1	0.94	11.8	2	2
23	44	F	12	155	115	2.3	17.9	0.01	1	14	1	G151R	APA	120	83	4.3	11.3	1.62	7	2	2
24	51	F	15	180	115	3.2	18.6	0.01	1	16	0	WT	APA	118	75	4.5	7.9	0.77	10.3	2	2

25	36	F	5	140	95	2.5	24.3	0.01	1	8	1	L168R	APA	113	64	4.7	8.2	0.42	19.5	2	2
26	41	M	6	180	130	3.4	19.2	0.03	1	12	1	L168R	APA	130	72	4	11.9	0.93	12.8	2	2
27	58	M	20	210	110	1.8	27.2	0.01	1	13	1	L168R	APA	118	63	4.4	5.6	2.72	2.1	2	2
28	33	M	3	199	121	3.1	23.7	0.462	1	16	1	G151R	APA	120	73	4	8.3	1.61	5.2	2	2
29	71	F	33	206	120	2.9	16.3	0.34	1	14	0	WT	APA	127	63	4.5	8.9	1.67	5.3	2	2
30	51	M	20	200	120	3.9	17.6	0.01	1	24	0	WT	APA	110	80	4.6	9.7	2.27	4.3	2	2
31	58	F	23	225	115	4	19.6	0.01	1	23	0	WT	APA	124	83	4.1	7	2.07	3.4	2	2
32	46	M	7	195	105	2.3	24.9	0.01	1	12	1	G151R	APA	113	78	4.2	7.7	1.09	7.1	2	2
33	56	F	20	200	110	2.4	16.3	0.01	1	18	1	L168R	APA	122	82	4.5	8.5	0.77	11	2	2
34	34	M	5	186	130	2.4	17	0.02	1	15	1	L168R	APA	122	65	4.4	9.5	0.69	13.8	2	2
35	44	F	5	160	102	3.6	17.8	0.01	1	9	1	G151R	APA	112	66	4	9.3	0.82	11.3	2	2
36	49	M	17	143	120	3.5	15.7	0.01	1	27	1	L168R	APA	133	95	4.3	6.9	1.22	5.7	1	2
37	62	M	22	220	120	3.4	19.7	0.15	1	17	1	G151R	APA	115	72	4.4	10.2	2.25	4.5	2	2
38	43	M	14	190	115	3.5	20.3	0.01	1	14	0	WT	APA	113	73	4.4	10.6	1.78	6	2	2
39	51	M	20	200	120	4.2	19.6	0.11	1	22	0	WT	APA	109	81	4.6	12	0.95	12.6	2	2
40	47	M	15	220	135	2.3	21.3	0.01	1	19	1	G151R	APA	125	82	4.6	9.8	1.98	4.9	2	2
41	50	M	11	153	110	3.2	23.1	0.01	1	8	0	WT	APA	118	80	4.5	13.2	0.65	20.3	2	2
42	71	F	40	190	110	4	23.1	0.01	1	9	1	G151R	APA	116	79	4.9	5.5	0.89	6.2	2	2
43	55	M	13	184	105	2.8	34.4	0.01	1	15	1	G151R	APA	129	70	4.5	6.8	2.25	3	2	2
44	49	M	14	170	110	3.3	21.8	0.01	1	18	1	G151R	APA	126	77	4.5	12.4	2.76	4.5	2	2
45	49	F	13	202	100	4.1	16.8	0.01	1	17	1	G151R	APA	148	94	5.1	10.9	2.31	4.7	1	2
46	50	M	19	180	100	2.1	23.1	0.03	1	15	1	G151R	APA	130	80	4.2	12.4	2.75	4.5	2	2
47	53	F	16	155	110	2.5	15.7	0.01	1	29	1	G151R	APA	117	69	4.1	5.2	0.49	10.6	2	2
48	25	F	2	165	110	4	19.7	0.01	1	13	1	L168R	APA	114	75	4.3	7.2	2.84	2.5	2	2
49	30	F	2	168	101	4.1	17.8	0.01	1	24	1	L168R	APA	120	79	4.3	12.4	1.84	6.7	2	2
50	61	F	17	180	110	4	19.8	0.01	1	14	1	G151R	APA	127	70	4.8	12.8	2.38	5.4	2	2
51	33	M	6	160	110	2.3	17.6	0.01	1	16	1	G151R	APA	121	62	4	11.2	0.77	14.5	2	2
52	28	F	6	180	110	3.1	24.9	0.01	1	10	1	L168R	APA	110	78	4.1	6.6	1.44	4.6	2	2
53	56	M	20	160	115	3.2	39.6	0.01	1	19	0	WT	APA	116	70	4.3	5.2	2.81	1.9	2	2
54	35	M	3	180	120	3	24.4	0.01	1	18	1	G151R	APA	121	79	4.2	5.9	0.53	11.1	2	2
55	63	M	33	185	122	2.9	15.9	0.01	1	12	1	G151R	APA	110	83	4.3	12.6	2.94	4.3	2	2
56	46	M	6	200	120	3.3	17.9	0.13	1	20	0	WT	APA	132	76	4.9	7.9	2.09	3.8	2	2
57	76	M	30	185	105	4.3	18.2	0.16	1	25	0	WT	APA	111	75	4	12.8	2.3	5.6	2	2

58	67	F	30	164	98	4.2	22.7	0.01	1	14	0	WT	APA	116	82	4.6	6.4	1.4	4.6	2	2
59	56	M	16	180	110	3.9	15.11	0.01	1	22	0	WT	APA	146	93	4.1	9.8	1.06	9.2	1	2
60	61	F	19	160	115	2.6	19.9	0.34	1	8	0	WT	APA	115	81	4.1	5.4	3.02	1.8	2	2
61	64	F	18	155	103	4.1	23.3	0.06	1	12	0	WT	APA	128	63	4	12.5	2.81	4.4	2	2
62	64	F	34	200	100	4.3	17.1	0.14	1	9	0	WT	APA	129	74	4.6	10.3	1.47	7	2	2
63	37	F	8	217	123	4.1	15.6	0.01	1	19	0	WT	APA	118	73	4	10.9	0.67	16.3	2	2
64	30	M	5	210	110	1.83	22	0.01	1	26	1	G151R	APA	120	73	4.1	11.5	2.91	4	2	2
65	48	F	8	215	110	3.8	21.4	0.01	1	25	1	G151R	APA	123	66	4.4	7.2	0.96	7.5	2	2
66	37	M	7	180	110	1.9	22.3	0.22	1	12	0	WT	APA	124	70	4.2	9.2	2.29	4	2	2
67	60	M	13	169	107	3.7	34.1	0.03	1	24	1	L168R	APA	122	72	4.1	6.2	0.37	16.8	2	2
68	58	M	20	160	100	3.6	3307	0.01	1	20	0	WT	APA	113	77	4.1	10.3	3.29	3.1	2	2
69	56	M	16	180	110	3.6	16.26	0.07	1	14	0	WT	APA	124	66	4.3	11.9	2.67	4.5	2	2
70	55	M	19	200	120	3	22.6	0.01	1	12	0	WT	APA	160	93	5	9.5	1.86	5.1	1	2
71	50	M	7	178	116	4.2	20.5	0.01	1	16	0	WT	APA	120	78	4.4	7.4	1.06	7	2	2
72	60	M	24	165	112	3.6	42.5	0.01	1	19	1	L168R	APA	117	74	4.2	7.4	2.27	3.3	2	2
73	41	F	11	192	110	3.4	17.2	0.04	1	14	1	G151R	APA	114	64	4.2	12.3	3.05	4	2	2
74	66	F	20	170	100	3.5	41.8	0.01	1	12	0	WT	APA	113	64	4.5	11.5	1.36	8.5	2	2
75	37	F	12	185	117	4.1	26.1	0.01	1	22	1	G151R	APA	121	71	4	10.6	0.8	13.3	2	2
76	41	M	5	170	100	2.2	22	0.11	1	15	1	G151R	APA	119	68	4.9	9.6	1.29	7.4	2	2
77	46	F	16	166	111	4.3	16.5	0.24	1	15	1	G151R	APA	114	70	4.1	11.9	0.85	14	2	2
78	55	M	20	175	110	3.2	39.2	0.05	1	18	0	WT	APA	135	73	4	11.4	2.79	4.1	2	2
79	56	M	15	162	105	2.8	48.2	0.01	1	9	1	L168R	APA	115	82	4.9	9.5	2.01	4.7	2	2
80	32	F	5	180	110	3.4	33.3	0.05	1	17	0	WT	APA	128	64	4.3	9.4	1.21	7.8	2	2
81	58	M	15	170	120	2.6	17.36	0.14	1	16	0	WT	APA	124	71	4.2	9.3	1.07	8.7	2	2
82	31	F	3	170	130	3.5	30.5	0.01	1	13	0	WT	APA	132	66	4.7	7	1.77	4	2	2
83	41	M	8	180	100	2.6	28.6	0.01	1	22	1	L168R	APA	118	60	5.8	8	1.01	7.9	2	2
84	30	F	3	220	130	2.3	24.1	0.14	1	15	0	WT	APA	153	100	4.6	9.6	1.11	8.6	1	2
85	44	M	10	160	110	4.5	16.4	0.35	1	24	0	WT	APA	110	71	4.6	7.3	0.8	9.1	2	2
86	56	F	19	167	106	3.9	23.1	0.04	1	11	0	WT	APA	112	63	4.1	10.3	2.81	3.7	2	2
87	46	M	12	170	110	2.1	33.6	0.01	1	20	1	L168R	APA	123	66	4.6	11.4	0.46	24.8	2	2
88	63	M	20	180	110	4.2	24.3	0.03	1	13	1	L168R	APA	119	67	4.1	10.5	1.79	5.9	2	2
89	33	F	2	145	98	2.3	23.4	0.16	1	10	0	WT	APA	121	62	4.2	7.1	0.39	18.2	2	2
90	38	M	2	169	118	3	38.1	0.01	1	13	0	WT	APA	116	60	4.1	10.2	2.05	5	2	2

91	49	F	17	162	106	3.2	19.3	0.13	1	18	1	L168R	APA	125	80	5	14.1	0.91	15.5	2	2
92	69	M	19	174	114	2.7	28.4	0.05	1	16	0	WT	APA	125	60	4	12.7	1.74	7.3	2	2
93	63	F	35	210	115	2.6	25.8	0.2	1	11	1	L168R	APA	114	66	4.4	6.9	1.31	5.3	2	2
94	30	F	2	157	111	2.5	20.5	0.39	1	10	0	WT	APA	128	72	4	9.2	1.89	4.9	2	2
95	48	F	9	222	102	4	19.2	0.12	1	20	0	WT	APA	114	72	4.5	12.2	2.5	4.9	2	2
96	43	F	10	175	110	4.4	17.9	0.06	1	19	1	G151R	APA	122	73	4.2	9	2.17	4.1	2	2
97	60	M	10	164	128	2.9	19.9	0.09	1	18	1	G151R	APA	121	82	4.1	10.7	0.91	11.8	2	2
98	45	M	3	143	105	3.3	24.4	0.18	1	9	1	L168R	APA	112	74	4.1	11.4	1.17	9.7	2	2
99	44	M	14	152	112	4.2	15.1	0.04	1	8	1	G151R	APA	128	63	4.2	9.1	1.91	4.8	2	2
100	58	M	15	155	110	3	23.2	0.01	1	13	1	G151R	APA	121	76	4.3	5.8	1.42	4.1	2	2
101	50	M	15	199	119	3.2	15.4	0.1	1	15	0	WT	APA	114	79	4.9	10.1	2.21	4.6	2	2
102	63	M	25	177	107	3.7	17.1	0.01	1	17	0	WT	APA	144	93	4.5	8.3	2.36	3.5	1	2
103	48	F	12	193	115	2.6	20.1	0.18	1	19	1	L168R	APA	123	81	4.3	8.4	3.07	2.7	2	2
104	29	F	2	151	113	3.5	23.5	0.24	1	12	1	L168R	APA	121	71	4.1	12.6	2.78	4.5	2	2
105	42	F	14	177	110	3	19.2	0.01	1	10	0	WT	APA	151	80	4.2	7.8	1.11	7	1	2
106	46	F	5	150	103	2.6	52.54	0.99	2	L17; R21	1	G151R	APA	133	75	4.6	11.8	1.22	9.6	2	2
107	30	F	1	177	115	3.2	19.6	0.04	1	9	0	WT	IHA	155	103	4	17.5	0.1	175	1	1
108	54	F	15	170	120	2.8	24.7	0.16	1	10	0	WT	IHA	170	120	2.7	30.5	0.15	203.3	0	0
109	66	F	30	185	100	2.9	33.5	0.1	1	22	0	WT	IHA	155	100	3.1	30.6	0.12	255	1	0
110	51	F	11	168	110	2.9	21.4	0.02	1	17	0	WT	IHA	158	95	4.2	16.4	0.1	164	1	1
111	57	M	14	205	120	2.2	20	0.14	1	16	0	WT	IHA	147	105	5.1	15.2	0.21	72.4	1	1
112	43	M	6	220	131	3.3	18.9	0.09	1	10	0	WT	IHA	151	110	3.4	21.6	0.08	270	1	0
113	61	M	24	175	105	3.3	18.6	0.11	1	18	0	WT	IHA	158	99	4	16.5	0.34	48.5	1	1
114	37	M	5	160	120	3	22.1	0.14	1	9	0	WT	IHA	160	100	4.1	15.8	0.15	105.3	1	1
115	41	M	11	220	120	2.4	23.9	0.02	1	21	0	WT	IHA	143	95	3.8	12.98	0.09	144.2	1	1
116	43	M	6	180	115	3.3	20.5	0.06	1	11	0	WT	IHA	150	100	5	18.3	0.13	140.8	1	1
117	63	M	26	160	110	2.8	23	0.17	1	14	0	WT	IHA	155	100	4	18.8	0.11	170.9	1	1
118	50	M	12	164	118	2.9	19	0.01	1	11	0	WT	IHA	154	90	4.5	18.6	0.38	48.9	1	1
119	57	F	35	170	106	2.5	37.2	0.01	1	17	0	WT	IHA	148	95	3.7	30.03	0.08	375.4	1	1
120	30	F	2	170	120	3.3	17.4	0.04	1	14	0	WT	IHA	150	100	4.1	16.2	0.12	135	1	1
121	44	F	13	150	110	3	40.3	0.06	1	15	0	WT	IHA	150	105	3.9	17.1	0.19	90	1	1
122	22	M	0	120	65	4.1	10.5	0.56	NF-A	38	0	WT	NF-A	125	69	3.8	12.5	1.09	11.47	-	-

123	50	F	7	145	90	4.6	11	1.1	SCS-A	42	0	WT	SCS-A	150	90	4	9.5	2	4.75	-	-
124	27	M	1	150	100	3.9	7.1	0.87	SCS-A	42	0	WT	SCS-A	148	95	4.5	8.3	0.66	12.57	-	-
125	37	M	7	147	95	4.2	6.2	0.28	CS-A	35	0	WT	CS-A	138	85	4	8.2	0.33	24.85	-	-
126	41	F	2	151	92	4.5	13.3	1.19	NF-A	41	0	WT	NF-A	140	80	3.9	15.1	1.64	9.21	-	-
127	48	F	12	160	100	4	8.6	0.66	NF-A	55	0	WT	NF-A	160	100	3.7	10.2	0.51	20	-	-
128	61	F	5	155	95	5	15.9	2.1	SCS-A	51	0	WT	SCS-A	150	95	4.8	12.2	1.59	7.67	-	-
129	37	M	0	115	62	3.8	8.2	0.88	NF-A	35	0	WT	NF-A	106	60	4.6	16.8	1.1	15.27	-	-

NF-A: Nonfunctional adrenal adenoma

CS-A: Adrenal adenoma associated with Cushing's syndrome

SCS-A: Adrenal adenoma associated with subclinical Cushing's syndrome

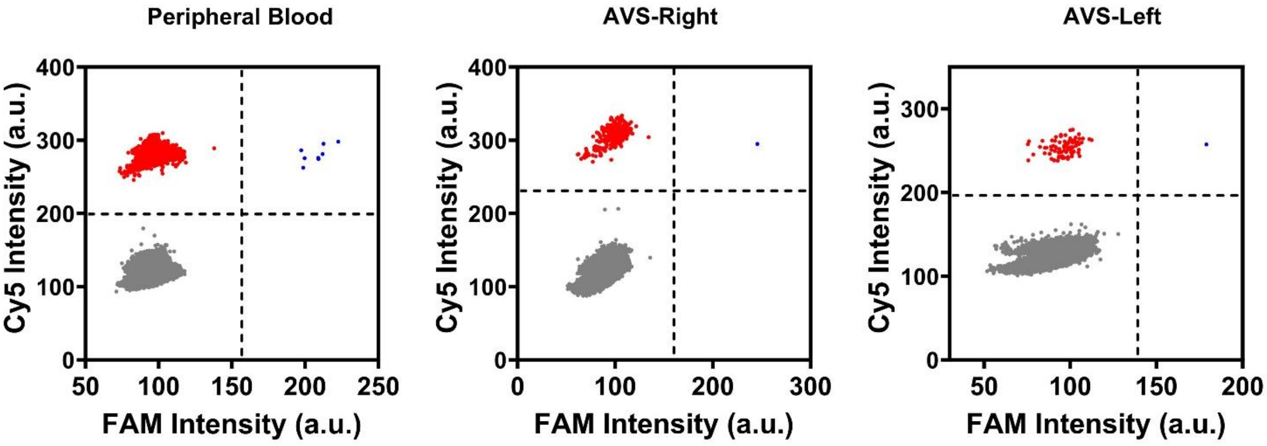

FIGURE S1 Two-dimensional ddPCR plots showing G151R (c.451G>A) variants in supernatants of peripheral blood, AVS-Right, and AVS-Left.
